# Drug Candidate BIO101 for Spinal Muscular Atrophy as Monotherapy or Combined With the Antisense Oligonucleotide ASO‐10‐27

**DOI:** 10.1002/jcsm.70104

**Published:** 2025-10-23

**Authors:** Cynthia Bézier, Steve Cottin, Parvin Nazari Hashemi, Mirella El Khoury, Zoé Clerc, Christine Balducci, Delphine Sapaly, Laure Weill, René Lafont, Stanislas Veillet, Pierre J. Dilda, Frédéric Charbonnier, Mathilde Latil, Olivier Biondi

**Affiliations:** ^1^ Université Paris Cité & INSERM UMR‐S1124 Paris Cedex 06 France; ^2^ Biophytis Sorbonne Université Paris Cedex 05 France; ^3^ Sorbonne Université Institut de Biologie de Paris‐Seine (BIOSIPE) & CNRS Paris France; ^4^ Laboratoire de Biologie de l'Exercice pour la Performance et la Santé (LBEPS), UMR, Université d'Evry, IRBA Université de Paris Saclay Evry‐Courcouronnes France

**Keywords:** 20‐hydroxyecdysone, combinatorial therapy, motor performance, motor units, mouse models, spinal muscular atrophy

## Abstract

**Background:**

Spinal muscular atrophy (SMA) is a neuromuscular disease caused by loss of survival of motor neuron (SMN) protein inducing progressive muscle weakness and atrophy due to motor neurons degeneration. Despite benefits of SMN restoration therapies in patients, motor defects are still persistent. We investigated the potential of BIO101, a new drug candidate promoting muscle growth by activating the protective arm of the renin‐angiotensin system through the MAS receptor, as monotherapy or in combination with the SMN‐based therapy ASO‐10‐27 (Nusinersen).

**Methods:**

BIO101 was administrated daily on severe or mild Taiwanese SMA mouse models or diluted in culture medium of SMA patient‐derived myoblasts. The BIO101 effects were evaluated on severe SMA mouse model in vivo (growth, survival and motor function), ex vivo (motor neuron, neuromuscular junction maturation, skeletal muscle phenotype) and on muscle SMN expression, while motor function effects were evaluated on mild SMA mouse model. The in vitro effects on proliferation, differentiation, metabolism and SMN expression of SMA patient‐derived myoblasts were analysed. Effects of the combination of BIO101 with ASO‐10‐27 were evaluated on severe SMA mouse model, in vivo and on tissular intracellular AKT signalling and SMN expression.

**Results:**

In severe SMA mice, BIO101 alone protected lateral motor neurons (+20%, *p* < 0.05), limited muscular atrophy (+30%, *p* < 0.01), accelerated maturation of muscular fibres (+70% for fast‐twitch muscles) and neuromuscular junctions (+50% of perforated clustering, *p* < 0.05) with more prominent effects on fast‐twitch muscles. Those adaptations led to an improvement of muscular function, significant at 7, 9 and 10 days post‐natal (+2‐fold for crossed squares and time of suspension, *p* < 0.01), which was also observed in mild SMA mice at 8 and 9 months of age (*p* < 0.01). Interestingly, BIO101 treatment also improved SMA patient‐derived myoblast differentiation (+20% myotube diameter and nuclei/myotube, *p* < 0.05) and anaerobic performances (ECAR, + 10%; *p* ≤ 0.05) without any impact on the proliferative state and aerobic capacities through MAS receptor activation. All BIO101 effects were independent of SMN protein expression. When combined with the ASO‐10‐27, BIO101 enhanced even more muscle resistance to fatigue (> 3‐fold over 27 days for time of suspension, *p* < 0.05) when compared with severe SMA mice treated with ASO‐10‐27 alone, without effects on survival through the activation of AKT intracellular pathway and independently of SMN protein expression.

**Conclusions:**

We showed that BIO101 constitutes an efficient SMN‐independent therapy to improve muscle performance in SMA, which could open new therapeutic avenues for patients in combination with SMN‐based therapies, or as monotherapy for less severe forms.

## 1 Introduction

Spinal muscular atrophy (SMA) is a neuromuscular disorder that stands as the leading genetic cause of death in childhood, affecting approximately 1 in 10 000 newborns [1]. The clinical phenotype encompasses a wide spectrum of severities, categorized in four types based on the age of onset and the motor symptoms. These range from type I, the most prevalent and severe form, to the adult form, the type 4 [2, S1]. SMA is caused by homozygous loss‐of‐function mutations of the telomeric *Survival of Motor Neuron 1* gene (*SMN1*) coding for the ubiquitously expressed SMN protein [3]. While patients possess at least one copy of the paralog gene *SMN2*, approximately 90% of its transcripts lack exon 7, resulting in a truncated and unstable SMNΔ7 protein [4, S2, S3]. Only 10% of the *SMN2* transcripts encode full‐length SMN protein, enabling viability but not fully compensating for the lack of *SMN1*. Thus, variation in genomic *SMN2* copy number and expression is inversely correlated to SMA severity [5].

The discovery of this genetic context has recently led to the approval of three therapies focusing on increasing SMN expression. These include (1) modulation of *SMN2* mRNA splicing using antisense oligonucleotide (ASO) (Spinraza, synonyms: nusinersen, ISIS 396443, ISIS‐SMN_Rx_, ASO‐10‐27) [6], (2) small molecule (Evrysdi, risdiplam) [7] and (3) viral vector‐based gene therapy to compensate the mutated *SMN1* gene (Zolgensma, onasemnogene abeparvovec) [8]. While these therapies demonstrate significant improvements in survival and motor function, none are considered curative. Their clinical efficacy varies depending on the therapeutic modality, as well as the age and severity of the patients [9, 10]. This supports the need to develop novel therapeutic strategies targeting residual pathophysiological alterations and persistent neuromuscular defects that cannot be fully reversed by improving SMN expression alone [11, S6]. Most of the new approaches under development are targeting the muscle, aiming to increase muscle mass through myostatin inhibition [12, S7–S9] or to improve muscle contractility using a fast skeletal muscle troponin activator [13]. However, these approaches do not comprehensively address the muscular function in its entirety, from the muscle itself to its environment, nor the broader multi‐tissue and metabolic dimensions. In SMA context, several alterations of the muscular metabolism have been reported, including glycolysis, mitochondrial function and vasculature defects, which contribute directly to muscle wasting, fatigue and disease severity. This highlights that metabolic dysregulation in muscle is both a consequence of SMN deficiency and a potential therapeutic target in SMA. To date, no drug candidates targeting muscle metabolism and function have been approved for the treatment of SMA.

In this context, 20‐hydroxyecdysone (20E), the most prevalent phytoecdysteroid, emerges as promising candidate to positively impact the SMA disease progression. Indeed, this molecule has a broad spectrum of pharmacological effects in mammals [14], such as stimulation of muscle anabolism [S10], promotion of angiogenesis [S11] and modulation of energetic metabolism [S12, S13], all of which are affected in SMA and play crucial roles in muscle function [15, 16]. 20E activates the MAS receptor, a key component of the renin‐angiotensin system, eliciting a rapid Ca^2+^ flux leading to AKT activation and increased protein synthesis in skeletal muscle cells [17, S15]. BIO101, a pharmaceutical‐grade preparation of 20‐hydroxyecdysone (> 97% purity), has been developed and demonstrated a favourable safety profile in adults, as well as beneficial effects on mobility in sarcopenic patients [18].

This study utilized myoblasts from type 2 SMA patients and severe (*Smn* ^Δ7/Δ7^; tg*SMN2* ^0/+^, two copies) and mild (*Smn* ^Δ7/Δ7^; tg*SMN2* ^+/+^, four copies) Taiwanese SMA mouse models [19] to investigate BIO101 effects from molecular and cellular to behavioural levels. Severe Taiwanese SMA mouse model is characterized by critical defect in growth and motor function leading to death at around 12 days post‐natal, while mild Taiwanese SMA mouse model is characterized by nonlethal distal necrosis and progressive motor neurons death (from 6 months of age) and motor defect (from 9 months of age). The results indicate that BIO101 monotherapy exerts beneficial effects on myoblasts from SMA patients, as well as on the entire motor unit of severe SMA mice (*Smn* ^Δ7/Δ7^; tg*SMN2* ^0/+^), leading to an enhancement of muscle function. These effects are more pronounced in mild SMA mice (*Smn* ^Δ7/Δ7^; tg*SMN2* ^+/+^). Interestingly, when combined with a single intracerebroventricular injection of ASO‐10‐27, BIO101 considerably improves muscle function of severe SMA mice without significantly affecting others in vivo parameters. All BIO101‐induced benefits were independent of SMN expression, despite the expected activation of the AKT intracellular signalling. Altogether, these findings collectively support the consideration of BIO101 as a prime candidate for a wide application in SMA patients.

## 2 Materials and Methods

### 2.1 BIO101

BIO101, the Good Manufacturing Practice drug substance at pharmaceutical‐grade of 20‐hydroxyecdysone (≥ 97%), was from Patheon (Regensburg, Germany). For mice treatment, BIO101 was complexed with (2‐hydroxypropyl)‐β‐cyclodextrin) (H107, Sigma).

### 2.2 Mice and treatments

Animal handling and experimentation were performed in line with approved Institutional Animal Care and Use Committee protocols at the Paris‐Cité University (CEEA 34, agreement number C750607), following the national authority guidelines (Ministère de la Recherche et de la Technologie, France) on ethical treatment of laboratory animals based on European Union Directive 2010/63/EU (APAFIS#26872‐2019 062 115 564 717 v7). Transgenic mild Taiwanese SMA males (*Smn* ^Δ7/Δ7^; tg*SMN2* ^+/+^, FVB.Cg‐Smn1t^m1Hung^ Tg (SMN2)2Hung/J, strain #005058) [19] were obtained from the Jackson Laboratory and were crossed with either heterozygous *Smn* knock‐out females (*Smn* ^Δ7/+^), for generating severe Taiwanese SMA (FVB/NRj‐*Smn* ^Δ7/Δ7^; tg*SMN2* ^0/+^) and control mice (FVB/NRj‐*Smn* ^+/Δ7^; tg*SMN2* ^0/+^), or with heterozygous *Smn* knock‐out females having four copies of the human *SMN2* transgene (FVB/NRj‐*Smn* ^Δ7/+^; tg*SMN2* ^+/+^), for generating mild Taiwanese SMA (FVB/NRj‐*Smn* ^Δ7/Δ7^; tg*SMN2* ^+/+^) and control mice (FVB/NRj‐*Smn* ^Δ7/+^; tg*SMN2* ^+/+^).

The entire litter of severe SMA mice was assigned to one group of treatment at birth, but litters from the same female were systematically assigned to different groups of treatment. For mild Taiwanese SMA mice, animals were randomly assigned to different groups without sex discrimination. Severe Taiwanese SMA mice received either 50 mg/kg/day of BIO101 or the vehicle cyclodextrin (VH) in tap water by oral gavage from P0. When co‐treated, at P0, they received in addition a single intracerebroventricular injection, as previously described [20], of either 8 μg of ASO‐10‐27 (5′‐TCACTTTCATAATGCTGG‐3′) or six‐mismatch control (5′‐TTAGTTTAATCACGCTCG‐3′) produced by Eurogentec as previously described [21, S16]. Mild Taiwanese SMA mice received either only water (VH) or 50 mg/kg/day of BIO101 directly diluted in the water feeder from 6 to 9 months of age. For all molecular and tissular analyses, mice were anaesthetised at 10.5 days or 9 months of age by pentobarbital administration (EXAGON®) at 1 μL/g 1 h after the last BIO101 or VH treatment.

### 2.3 Motor Behaviour Assays

The open‐field and grip tests were performed as previously described [22], and details of these methods are described in the Supplemental Methods.

### 2.4 NMJ, Motor Neuron and Muscle Histology

For neuromuscular junction and motor neuron analysis, dissected tissues were fixed 24 h in 4% paraformaldehyde, rinsed in PBS and cut in free‐floating sections with a vibratome (75‐μm‐thick for muscles, 50‐μm‐thick for spinal cord; VT‐1000S, Leica Microsystems SAS). For muscle histology and typology and for spinal cord vascularization analysis, dissected tissues were individually frozen into −80°C isopentane solution, and then sectioned in a cryostat (CM3050 S, Leica Microsystems SAS) into 10‐μm‐thick sections. The haematoxylin and eosin (HE) staining of muscle fibres was performed as previously described [22]. The detailed methods for immunohistochemistry analysis and the antibodies used are described in the Tables S1 and S2 of Supplemental Methods.

All images were acquired with a Zeiss Z1 AxioObserver microscope (ZEN 2.3 software) using an ORCA Fusion Camera (C14440‐20UP, Hamamatsu) for fluorescent images and an RGB AxioCam ICC 1 camera (Zeiss) for colorimetric images. All analyses were done using the ImageJ software (National Institutes of Health).

### 2.5 High‐Performance Liquid Chromatography (HPLC)

BIO101 was quantified in plasma collected intracardially the day of sacrifice after liquid chromatography separation on a 1260 Infinity LC system (Agilent technologies) followed by quantitative mass spectrometry performed on a QQQ‐6420 MS/MS (Agilent Technologies) with electrospray positive ionization (API‐ES). Details of these methods are described in the Supplemental Methods.

### 2.6 Protein Extraction and Western Blot Analysis

Frozen tissues were dissolved in blending buffer using metal beads (69 989, Qiagen) and a TissueLyser MM300 (Qiagen). Protein samples (30 μg) were mixed with Laemmli sample buffer (161‐0747, Biorad), denatured at 95°C, and electrophoresed on a 12.5% polyacrylamide gel. Samples were transferred to nitrocellulose membranes using the Trans‐Blot Turbo transfer system (1 704 158 and 1 704 150, Biorad). The blot was blocked in 5% milk powder in PBS with 0.1% Tween‐20 for 1 h, then incubated with primary antibodies (Table S3) diluted in TBS with 0.1% Tween‐20 and 5% bovine serum albumin (24 h, 4°C). After washes, the blot was incubated with secondary antibodies (Table S3) diluted in TBS with 0.1% Tween‐20 and 5% bovine serum albumin (1 h, RT). Revelation was performed using SuperSignal West Pico PLUS chemiluminescent substrate (34 577, Thermo Fisher Scientific) and imaged using ImageQuant LS4000 (GE Healthcare Bio‐Science).

### 2.7 Culture Cell, Treatment and Metabolic Assay

Human immortalized myoblasts from one type 2 SMA patient were provided by Institute of Myology (Paris) under a non‐profit‐to‐non‐profit uniform biological material transfer agreement (UBMTA). Cells were cultured on 0.01% gelatin‐coated flasks in a growth medium (GM; Table S3) at a seeding density of 5 × 10^3^ cells/cm. For proliferation assay, GM was implemented with Hoechst 33342 (1 μg/mL, H1399, Thermo Fisher Scientific) and cells were automatically scored using the ImageXpress Pico (Molecular Devices) before and after 48 h of treatment with BIO101 (5 μM), the Mas receptor antagonist A779 (PP‐H6705‐00, Bio‐Techne; 10 μM) or both.

For differentiation, cells were plated at 40 × 10^3^ cells/cm on Matrigel (356 237, Corning BV) in GM, and when cells reached ~90% confluency, medium was switched to differentiation medium (DM; Table S3) with BIO101 (5 μM), A779 (10 μM) or both. Cells were either tested for the metabolic assay at 2 days of differentiation using Seahorse XFe24 FluxPaks (102 340‐100, Agilent Technologies) and XF Cell Mito Stress Test (103 015‐100, Agilent Technologies) according to manufacturer's instructions (Table S5), or fixed (4% paraformaldehyde, 20 min, RT; 10 099 464, VWR) at 2 or 6 days of differentiation, permeabilized (0.5% Tween‐20 in TBS, 30 min, RT) and stained with anti‐MF20 antibody (1:40, DSHB; 0.5% Tween‐20 and 8% donkey serum in TBS, 1 h, 4°C), Hoechst 33342 and donkey anti‐mouse Alexa Fluor 488 antibody (1:400, 715‐545‐150, Jackson ImmunoResearch; 0.5% Tween‐20 and 4% donkey serum in TBS, 1 h, RT) for assessing myotube diameter and nuclei number per myotube.

Additional details are presented in the Supplemental Methods.

### 2.8 Statistical Analysis

Following the instructions from ARRIVE2.0 guideline and the national ethical recommendation, we determined the minimum number of animals per group needed to achieve 85% of statistical power using the G*Power 3.1.9.7 calculator with muscle capacity as outcome measure for in vivo evaluations, and the myofiber cross‐section‐area for ex vivo evaluations. For survival, Kaplan–Meier survival curves were generated and followed by the log‐rank test for statistical significance. Following recommendations, in vivo data are represented as mean ± standard error of the mean, while ex vivo data are represented as mean ± standard deviation. For multiple group comparisons, statistical significance was determined using a Kruskal–Wallis test with post hoc Dunn's test or a two‐way ANOVA with post hoc Sidak test or a mixed model of repeated measures with post hoc Sidak test. For two group comparisons, statistical significance was determined with nonparametric Mann–Whitney test or *t*‐test according to normal distribution of data evaluated by Kolmogorov–Smirnoff test. Statistical analyses and graphs were performed with the GraphPad Prism version 8.

## 3 Results

### 3.1 BIO101 Chronic Treatment Improves Muscle Function in Severe and Mild Taiwanese SMA Mouse Models

**FIGURE 1:**
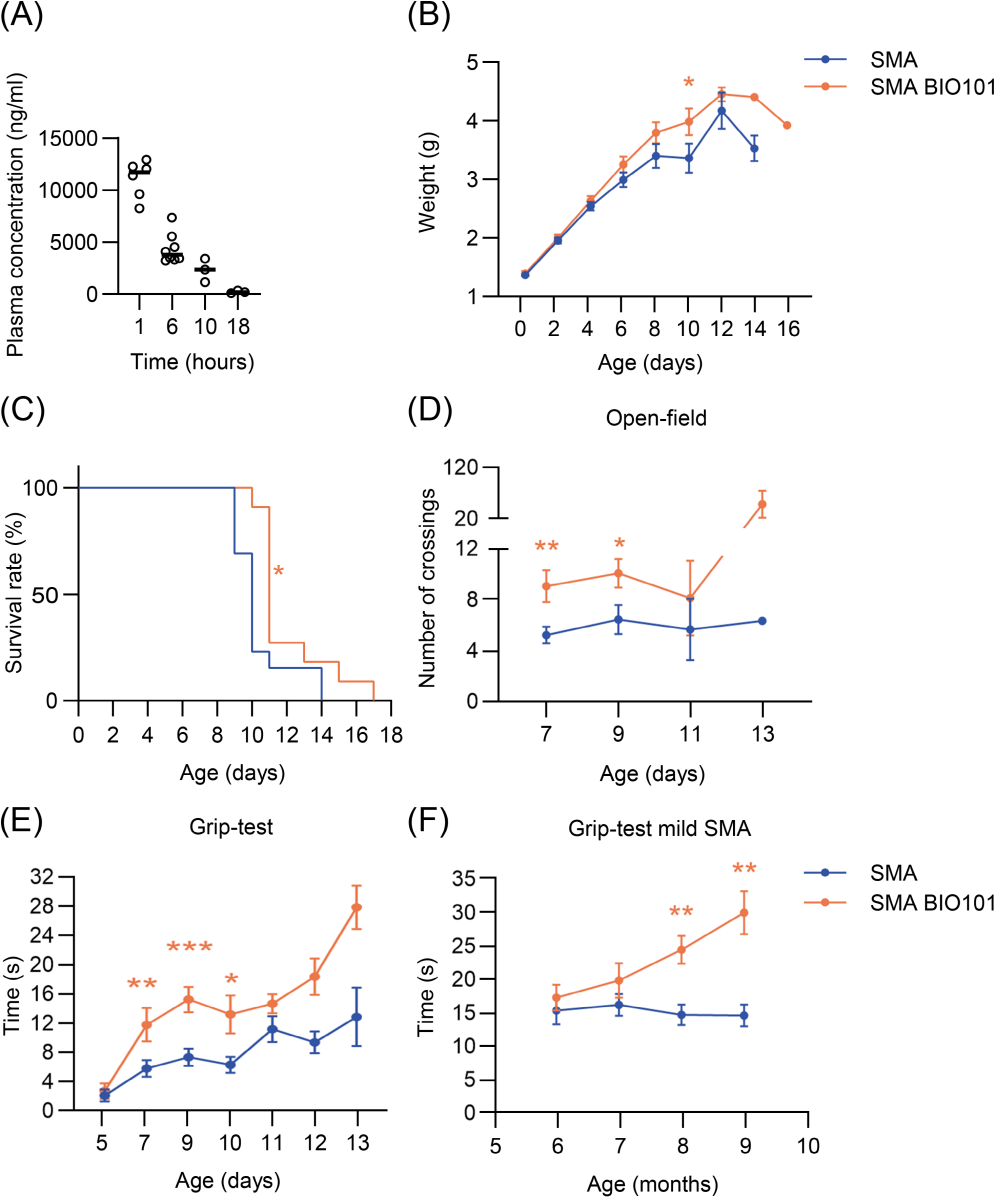
Effects of BIO101 on severe and mild Taiwanese SMA mouse phenotypes. (A) Evaluation of BIO101 plasma concentration of control mice after a single oral gavage with 50 mg/kg of BIO101 at P10.5 (*n* = 6 at 1 h; *n* = 8 at 6 h; *n* = 3 at 10 h; *n* = 3 at 18 h). (B) Weight curves of vehicle‐ or BIO101‐treated severe SMA mice (*n* = 25 and 19 mice respectively). (C) Survival curves of vehicle‐treated (*n* = 13 mice, median survival of 10 days) or BIO101‐treated (*n* = 11 mice, median survival of 10 days) severe SMA mice. Longitudinal recording of the total number of squares crossed (open‐field test, to evaluate moving capacity) (D) by vehicle‐ or BIO101‐treated severe SMA mice and of the maximal time of hind‐limb suspension (grip‐test, muscle fatigue) of vehicle‐ or BIO101‐treated severe (*n* = 25 and 16 mice respectively) (E) and mild (*n* = 18 and 15 mice respectively) (F) SMA mice. Data are represented as mean ± SEM with *p < 0.05 and **p < 0.01 by (B, D and E) unpaired *t*‐test at each age or (C) log‐rank test or (F) mixed model of repeated measures with a Sidak post hoc tests.

Based on a previous study, the bioavailability of the pharmaceutic grade preparation of 20‐hydroxyecdysone (> 97%), BIO101, was assessed after a single bolus at 50 mg/kg in post‐natal mice at 10.5 days of age, the late‐symptomatic stage in severe Taiwanese SMA mouse model (Figure 1A). We noted a progressive decay in its plasmatic concentration until 18 h post‐treatment, confirming the need of daily treatment with BIO101.

On Taiwanese SMA mouse model, we noted a negligible benefit on growth, notably from P6 to P10, and a nonsignificant shift of the final drop beyond P12 in BIO101‐treated severe SMA mice when compared with vehicle‐treated severe SMA mice (Figure 1B). Consistently, we observed a clinically nonrelevant gain in survival, with average survival increase by 2.5 days in BIO101‐ (13 days) compared with vehicle‐treated SMA mice (10.5 days; log‐rank *p = 0.0133; Figure 1C).

However, BIO101 treatment increased moving capacities (open field) with significant differences at P7 and P9 (*p* ≤ 0.05), and even more the resistance to muscular fatigue (grip test) with significant differences at P7 and P10 (*p* ≤ 0.05) in severe SMA mice (Figure 1D,E), and a marked improvement over P11.

We next determined if the motor‐specific effect of BIO101 could also be observed in the adult mild Taiwanese SMA mouse model (Smn^Δ7/Δ7^; tg*SMN2* ^+/+^). When treated daily during the symptomatic phase from 6 to 9 months of age [23, S18], we observed a constant improvement in time of suspension only for BIO101‐treated mild SMA mice (Figure 1F). Collectively, our results showed that in Taiwanese SMA mouse models, BIO101 treatment can specifically enhance muscular performances on a wide range of severities and ages, even when administered after the onset of motor defects.

### 3.2 BIO101 Protects Motor Neurons and Improves Spinal Cord Vascularization in Severe Taiwanese SMA Mouse Model.

**FIGURE 2:**
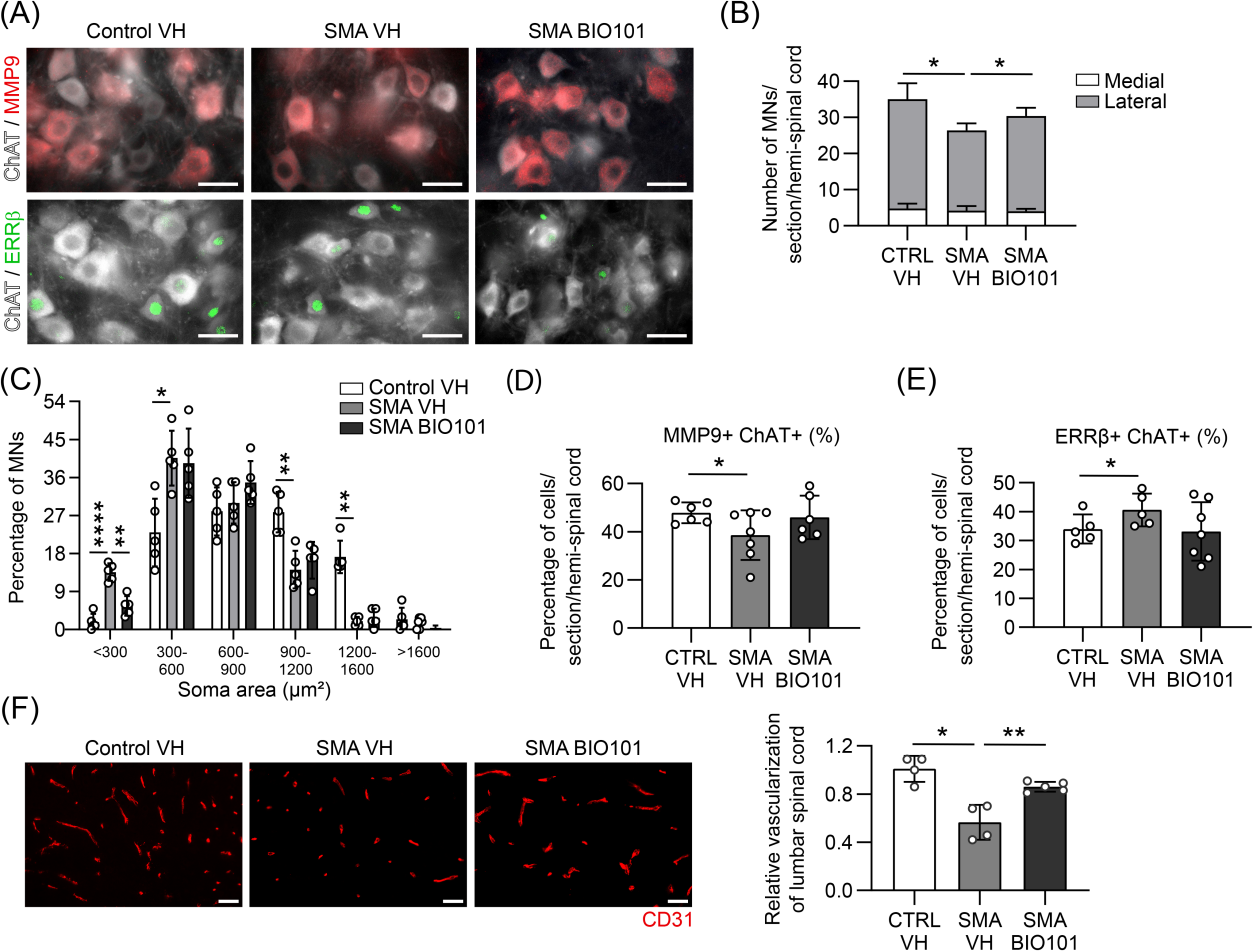
Effects of BIO101 on spinal cord in severe Taiwanese SMA mouse model. (A) Immunofluorescence directed against choline acetyltransferase (ChAT; motor neuron marker, white), oestrogen‐related receptor β (ERRβ; slow‐motor neuron marker, green) and matrix metallopeptidase 9 (MMP9; fast‐motor neuron marker, red; scale bar, 50 μm; 200× magnification) on 50‐μm‐thickness cross‐sections of spinal cord from vehicle‐treated control compared with vehicle‐ and BIO101‐treated severe SMA mice. (B and C) Quantification of the total number of ChAT+ cells depending on their localization (B), and of the distribution of their soma area (C, *n* = 5 mice in each group). (D and E) Quantification of the percentage of MMP9+ ChAT+ (D, *n* = 5 vehicle‐treated control mice, *n* = 6 mice in other groups) or ERRβ+ ChAT+ (E, *n* = 7 BIO101‐treated severe SMA mice, *n* = 6 mice in other groups) cells over total ChAT+ cells, in 50‐μm‐thick slice of lumbar spinal cord of vehicle‐treated control mice compared with vehicle‐ or BIO101‐treated severe SMA mice at P10.5. (F) Immunofluorescence of CD31 and quantitative analysis of the capillary density in 10‐μm‐thick slice of lumbar spinal cord of vehicle‐treated control mice (*n* = 4) compared with vehicle‐ or BIO101‐treated severe SMA mice at P10.5 (*n* = 4 and *n* = 5 mice respectively; scale bar, 20 μm; 200× magnification). Data are represented as mean ± SD with *p < 0.05; **p < 0.01, ****p < 0.0001 for a comparison between the two indicated groups or with vehicle‐treated control mice by (B, D, E and F) unpaired nonparametric Mann–Whitney tests or (C) two‐way ANOVA with a Sidak post hoc tests.

The specific enhancement of motor function with BIO101 treatment in severe and mild Taiwanese SMA mouse models led us to question the neuroprotection of different populations of motor neuron (MN). BIO101 treatment significantly limited the loss of absolute lateral MNs (+19%; *p* ≤ 0.05; Figure 2A,B) in BIO101‐treated severe SMA mice compared with vehicle‐treated severe SMA mice, with a significant decrease in the percentage of small MNs (< 300 μm^2^; Figure 2C). No effect of BIO101 treatment was observed on absolute number and size of MNs for control mice (Figure S1A,B). We further quantified the proportion of double‐positive immunostained cells for choline acetyltransferase (ChAT) and either oestrogen‐related receptor β (ERRβ) or matrix metallopeptidase 9 (MMP9), known to be markers of slow and fast MNs, respectively [24, S19]. We noted a significant decrease in the percentage of MMP9+ MNs (−20%; *p* ≤ 0.05) associated with a significant increase in the percentage of ERRβ+ MNs (+20%; *p* ≤ 0.05) in untreated severe SMA mice when compared with control mice, suggesting a fast‐specific degeneration in this model. A complete restoration of the proportion of fast over slow MNs was observed in BIO101‐treated severe SMA mice (Figure 2D,E) without any change in the total amount of slow MNs (Figure S1C), suggesting a specific neuroprotection of fast MN population.

In control mice, if BIO101 treatment did not modify the proportion of ChAT+ MMP9+ (Figure S1D), it induced a significant decrease in the proportion (−23%; *p* ≤ 0.05) of ChAT+ ERRβ+ cells (Figure S1E), suggesting an increase of a ChAT+ ERRβ‐MMP9‐ cells subpopulation after treatment. Importantly, those neuronal effects of BIO101 are associated with a significant limitation of the defect in lumbar spinal cord vascularization, assessed by the surface quantification occupied by vessels in spinal cord section, observed in vehicle‐treated severe SMA mice (+50%; *p* ≤ 0.05; Figure 2F,G), without any effect in control mice (Figure S1F).

All those results support the hypothesis that BIO101 has a specific effect on MN and can protect sensitive and fast MN subpopulations in severe Taiwanese SMA mouse model, possibly through the improvement of the lumbar spinal cord vascularization.

### 3.3 BIO101 Preferentially Improves the Maturation of Fast Muscles and Their NMJ in Severe Taiwanese SMA Mouse Model

We then evaluated the muscle‐specific adaptations of the neuromuscular maturation, known to be a critical SMA feature linked to disease progression and functional defects [25, 26]. As previously described, we compare three differently affected muscles of the calf [S22]: the fast‐twitch flexor *tibialis*, fast‐twitch extensor *plantaris* and slow‐twitch extensor *soleus* muscles and evaluated BIO101 effects on neuromuscular junctions (NMJ) and muscle maturation of these three muscles.

**FIGURE 3:**
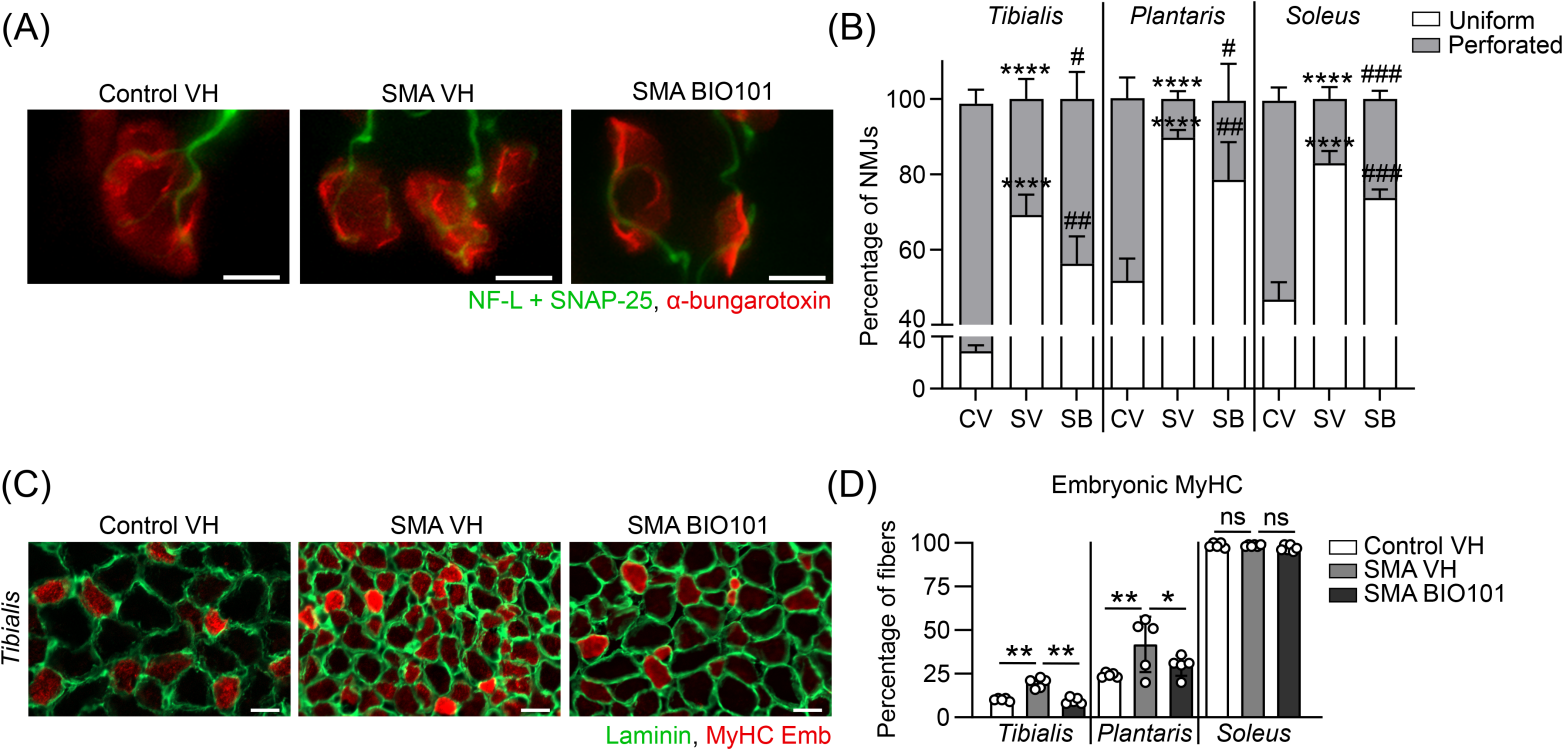
Effects of BIO101 on neuromuscular junction and muscle maturation in severe Taiwanese SMA mouse model. (A and B) Immunofluorescence of neurofilament L (NF‐L) and synaptosomal‐associated protein 25 (SNAP‐25) to label the pre‐synaptic motor nerve terminals (green), and with α‐bungarotoxin to label post‐synaptic side of neuromuscular junction (NMJ; red; scale bar, 20 μm; 200× magnification) (A) and quantification of the percentage of uniform and perforated NMJ over total NMJ (B) in the *tibialis*, *plantaris* and *soleus* muscles of vehicle‐treated control mice compared with vehicle‐ or BIO101‐treated severe SMA mice at P10.5 (*n* = 5 mice in each group). (C and D) Immunofluorescence of embryonic myosin heavy chain (MyHC; scale bar, 20 μm; at 200× magnification) (C) and quantitative analysis of their percentage over total number of myofibers (D) in the *tibialis*, *plantaris* and *soleus* muscles of vehicle‐treated control mice compared with vehicle‐ or BIO101‐treated severe SMA mice at P10.5 (*n* = 5 mice in each group). Data are represented as mean ± SD with *p < 0.05, **p < 0.01, and ****p < 0.0001 for a comparison between the two indicated groups or with vehicle‐treated control mice; and #*p* < 0.05, ##*p* < 0.01, and ###*p* < 0.001 for a comparison with vehicle‐treated severe SMA mice by (B) two‐way ANOVA with a Sidak post hoc tests or (D) unpaired nonparametric Mann–Whitney tests*.*

For NMJ, only innervated NMJs were analysed as showed in the *tibialis* of vehicle‐treated control mice (CV) compared with vehicle (SV)‐or BIO101‐treated (SB) severe SMA mice (Figure 3A). BIO101 treatment limited the delay of maturation of NMJs with a significant increase in the proportion of the perforated clustering [+42% (*p* ≤ 0.05) in the *tibialis*, +160% (p ≤ 0.05) in the *plantaris*, and +56% (*p* ≤ 0.001) in the *soleus*] and a concomitant decrease in the proportion of uniform clustering (Figure 3A,B) in severe SMA mice. No significant difference was observed in BIO101‐treated control mice despite muscle‐specific trends (Figure S2A).

For myofibers, we analysed the proportion of embryonic Myosin Heavy Chain isoform (MyHC, embryonic fibres) (Figure 3C,D) in the same muscles. We observed that BIO101 limits the expected delay in maturation of both fast‐twitch *tibialis* (−92%; *p* ≤ 0.01) and *plantaris* (−73%; *p* ≤ 0.01) muscles of severe SMA mice (Figure 3C,D), without any modification in the slow‐twitch *soleus* muscle (Figure 3D). In control mice, BIO101 treatment unexpectedly increased the percentage of embryonic MyHC positive fibres only in the fast‐twitch *tibialis* and *plantaris* muscles (+32% in the *tibialis* and +17% in the *plantaris*; Figure S2B).

Thus, we noted context‐specific effects of BIO101 accelerating the maturation of the NMJ and myofibers in SMA muscles and favouring immature neuromuscular junctions and myofibers in control muscles.

### 3.4 BIO101 Preferentially Improves Fast‐Muscle Phenotype and Vasculature in Severe Taiwanese SMA Mouse Model Through SMN‐Independent Mechanism

Then, we investigated whether BIO101‐induced improvements in motor neuron survival and neuromuscular maturation could be associated with muscular adaptations.

**FIGURE 4:**
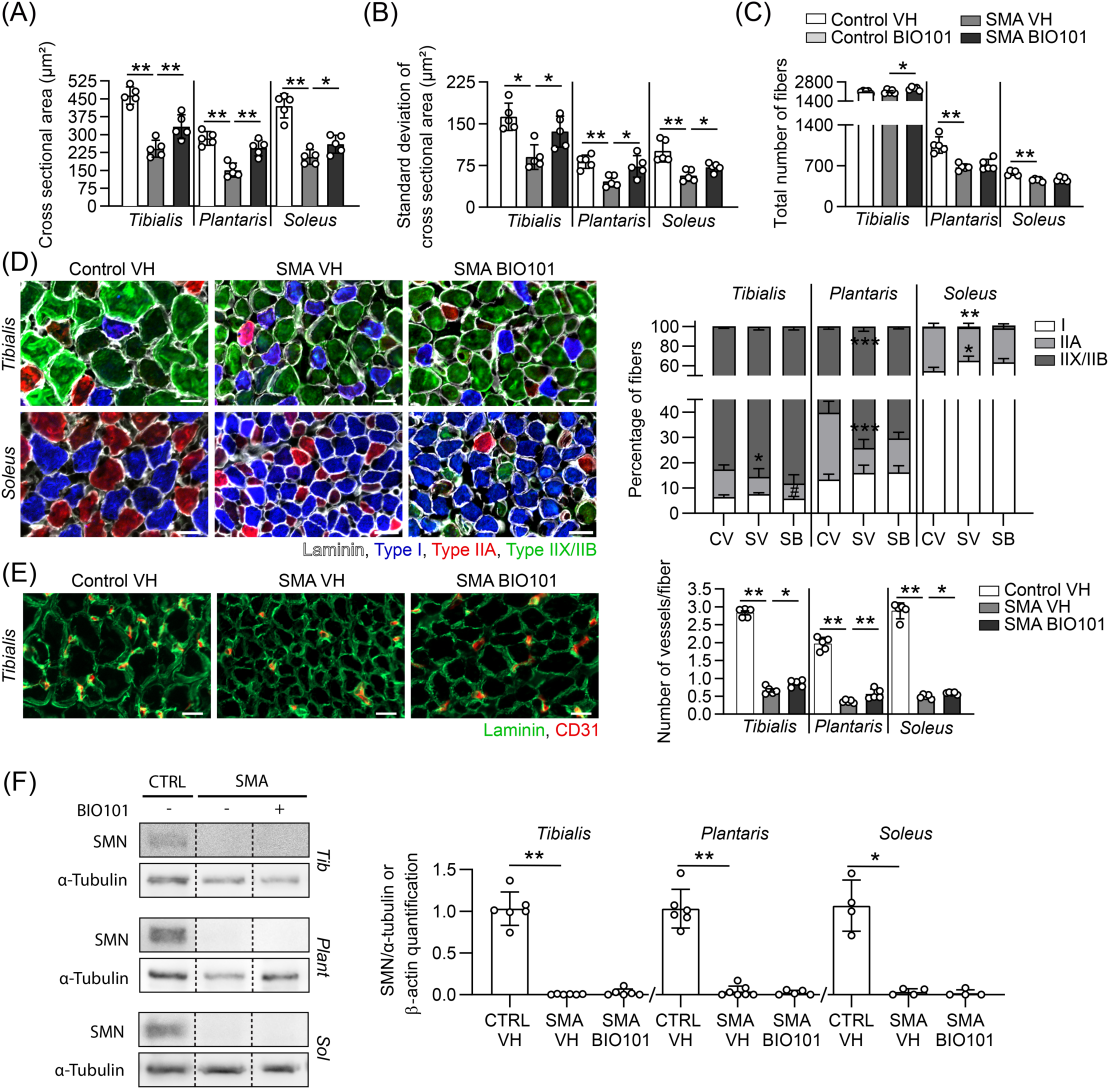
Effects of BIO101 on muscle phenotype in severe Taiwanese SMA mouse model. (A, B and C) Quantitative analysis of the mean of cross‐sectional area of fibres (A), their standard deviation (B) and the total number of fibres (C) in the *tibialis*, *plantaris* and *soleus* muscles of vehicle‐treated control mice compared with vehicle‐ or BIO101‐treated severe SMA mice at P10.5 (*n* = 5 mice in each group). (D) Immunofluorescence of laminin and adult type 1 (blue), type 2A (red) and type 2X/2B (white) isoforms of Myosin Heavy Chain and quantitative analysis of their percentage over the total number of myofibers in the *tibialis*, *plantaris* and *soleus* muscles of vehicle‐treated control mice (CV) compared with vehicle‐ (SV) or BIO101‐treated (SB) severe SMA mice at P10.5 (*n* = 5 mice in each group). (E) Immunofluorescence of CD31 and laminin and quantitative analysis of the number of vessels per muscle fibre in the *tibialis, plantaris* and *soleus* muscles of vehicle‐treated control mice compared with vehicle‐ or BIO101‐treated severe SMA mice at P10.5 (*n* = 5 mice in each group) (scale bars, 20 μm; 200× magnification). (F) Western Blot analysis and quantification of SMN expression in the hindlimb *tibialis*, *plantaris* and *soleus* muscles of vehicle‐treated control mice compared with vehicle‐ or BIO101‐treated severe SMA mice (*n* = 6 for *tibialis* and *plantaris*; *n* = 4 for *soleus*; Lanes were run on the same gel but were noncontiguous). Data are represented as mean ± SD with *p < 0.05 and **p < 0.01 for a comparison between the two indicated groups or with vehicle‐treated control mice, and #*p* < 0.05 for a comparison with vehicle‐treated severe SMA mice by (A, B, C, E and F) unpaired nonparametric Mann–Whitney tests or (D) two‐way ANOVA with a Sidak post hoc tests*.*

At P10.5, we observed a significant limitation of muscle atrophy (MA) in all studied muscles of severe SMA mice (Figure S3A and Figure 4A), with a concomitant enhancement of the variation among fibre sizes (standard deviation of fibre size per muscle, VCSA) (Figure S3A and Figure 4B). Interestingly, those effects were more prominent on the fast‐twitch tibialis (MA + 20%, VCSA +30%, *p* ≤ 0.01) and *plantaris* (MA + 33%, VCSA +30%, *p* ≤ 0.01) muscles than on the slow‐twitch *soleus* (MA + 13%, VCSA +15%, *p* ≤ 0.05) muscle (Figure S3A and Figure 4A,B). Moreover, in control mice, BIO101 treatment induced a hypertrophy and increase in VCSA in the fast‐twitch *tibialis* muscle only (Figure S3B,C).

In addition, BIO101 treatment significantly increased the total number of fibres per muscle only in the *tibialis* of severe SMA (+11%; *p* ≤ 0.01; Figure 4C) and control mice (+20%; *p* ≤ 0.01; Figure S3D), but failed in limiting SMA‐induced aplasia in the *plantaris* and the *soleus*; Figure 4C).

Furthermore, we looked at the muscle typology adaptations in response to treatment by quantifying the proportion of adult MyHC isoforms. If BIO101 treatment did not restore the altered proportion in slow‐twitch type I, intermediate type IIa and fast type IIx/D‐IIb positive myofibers observed in vehicle‐treated severe SMA mice compared with control mice, the treatment induced fast‐twitch muscle‐specific adaptations in both control and severe SMA mice (Figure 4D), without any modification of the slow‐twitch *soleus* (Figure 4D; Figure S3E).

Our results suggest that muscular BIO101 effects favour growth, through the increase in myofiber diameter and/or number according to the cellular context and facilitate intermediate and fast muscle typology whatever the conditions.

Interestingly, those muscular adaptations with BIO101 treatment were associated with a significant limitation of the vascular alteration, assessed by the number of vessels per myofiber, observed in vehicle‐treated severe SMA mice with more prominent effects in the fast‐twitch muscles [+29% (*p* ≤ 0.05) in the *tibialis*, and +58% (*p* ≤ 0.01) in the *plantaris*] than in the slow‐twitch *soleus* muscle (+20%; *p* ≤ 0.05; Figure 4E). In control mice, we also noted a specific fast‐twitch muscle improvement of the vasculature (*tibialis*: +11%; *plantaris*: +22%; *p* ≤ 0.05; Figure S3F).

We then questioned whether those BIO101‐induced benefits could rely on modulation in SMN protein expression. After confirming that the BIO101‐specific MAS receptor is well expressed in all studied muscles (Figure S3G) and not altered by chronic treatment (Figure S3H), we did not detect any effect of this chronic treatment on SMN protein expression by western blot in the three studied muscles (Figure 4F), demonstrating that all the muscular benefits observed in our study are independent of SMN protein expression.

### 3.5 BIO101 Accelerates SMA Patient‐Derived Myoblast Differentiation and Favour Anaerobic Metabolism

**FIGURE 5:**
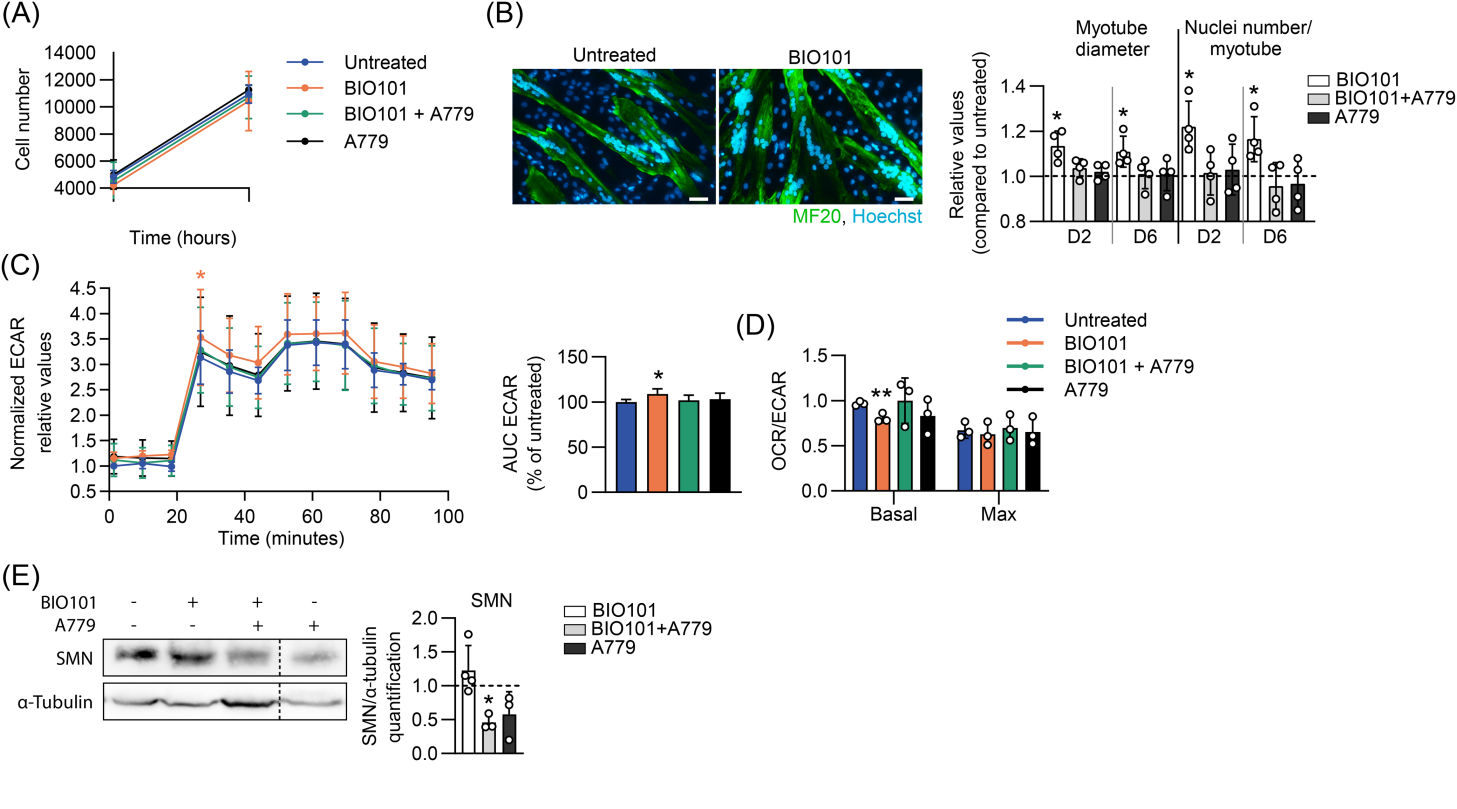
Effects of BIO101 on SMA patient‐derived myoblasts. (A) Quantification of the total number of cells before and 48 h after the treatment with BIO101 ± the specific inhibitor of MAS‐receptor (A779) in a culture of myoblasts derived from type 2 SMA patient in proliferation medium. (B) Immunofluorescence directed against the Myosin Heavy Chain common sequence to all isoforms (MF20, green) and DNA staining (Hoechst, blue) and quantitative analysis of myotube diameters and number of nuclei per myotube derived from type 2 SMA patient treated with BIO101 ± A779 at 2 (D2) and 6 days (D6) of differentiation (*n* = 4; scale bar, 50 μm; 200× magnification). (C and D) ECAR Mito stress test seahorse profiles and the analysis of their area under the curve (C) and OCR/ECAR ratio at basal (0 to 18 min) and maximal respiration (53 to 70 min) (D) in myotubes derived from type 2 SMA patient treated during 2 days of differentiation with BIO101 ± A779 (*n* = 3). (E) Western Blot analysis and quantification of SMN expression in myotube derived from type 2 SMA patient treated with BIO101 ± A779 at 6 days (D6) of differentiation (Lanes were run on the same gel but were noncontiguous). Data are represented as mean ± SD with *p < 0,05 and **p < 0.01 for a comparison with vehicle untreated cells by unpaired nonparametric Mann–Whitney tests (A, B, D and E) at 48 h or (C for AUC) for separated days of differentiation or (C, curves) at each time point.

This led us to consider the potential of BIO101 to positively affect muscular SMA patient cells. Although BIO101 did not affect the proliferation of SMA myoblasts over 48 h of culture (Figure 5A), we noted a significant increase in both myotube diameter (+14%; *p* ≤ 0.05) and number of nuclei per myotube (+22%; *p* ≤ 0.05) at 2 and 6 days of differentiation when compared with untreated SMA myotubes (Figure 5B). Associated with the decrease in *MyoD* and *Myogenin* mRNA expression, two main myogenic factors, at 2 days of differentiation prior to normalization at 6 days of differentiation (Figure S4A), BIO101 seems to accelerate the human myoblast differentiation through fusion process. Importantly, all BIO101 effects on myoblast differentiation were abolished by the specific MAS receptor antagonist A779, while the antagonist alone did not display any effect on myotube fate (Figure 5B and Figure S4A), confirming that BIO101 exerts its effects through MAS receptor signalling.

We then assessed the changes in energy metabolism of SMA patient‐derived myotubes at 2 days of differentiation, using Seahorse MitoStress test. If no change was observed on the oxygen consumption rate (OCR) under different conditions of mitochondrial stress (Figure S4B,C), we noted an increase in the extracellular acidification rate (ECAR) after the inhibition of the respiratory chain, at 30 min (+10%; *p* ≤ 0.05; Figure 5C,D). By reporting the OCR to the ECAR, our results support an enhancement of anaerobic catabolism of glucose in SMA myotubes under BIO101 treatment (Figure 5D), which was not observed in A779 (Figure 5C,D), supporting a MAS‐specific effect of BIO101 in favour of fast‐twitch myotube energy metabolism.

Finally, as observed in severe SMA‐like mice, BIO101‐induced benefits were independent of SMN protein expression in human myotubes after 6 days of differentiation (Figure 5E). Interestingly, the inhibition of MAS receptor with A779 induced a significant decrease in SMN expression when combined with BIO101 and a trend to decrease alone, suggesting a potent role of basal MAS receptor activity on SMN (Figure 5E).

### 3.6 BIO101 Potentiates the Benefits of ASO‐10‐27 Therapy on Motor Function in Severe Taiwanese SMA Mouse Model

**FIGURE 6:**
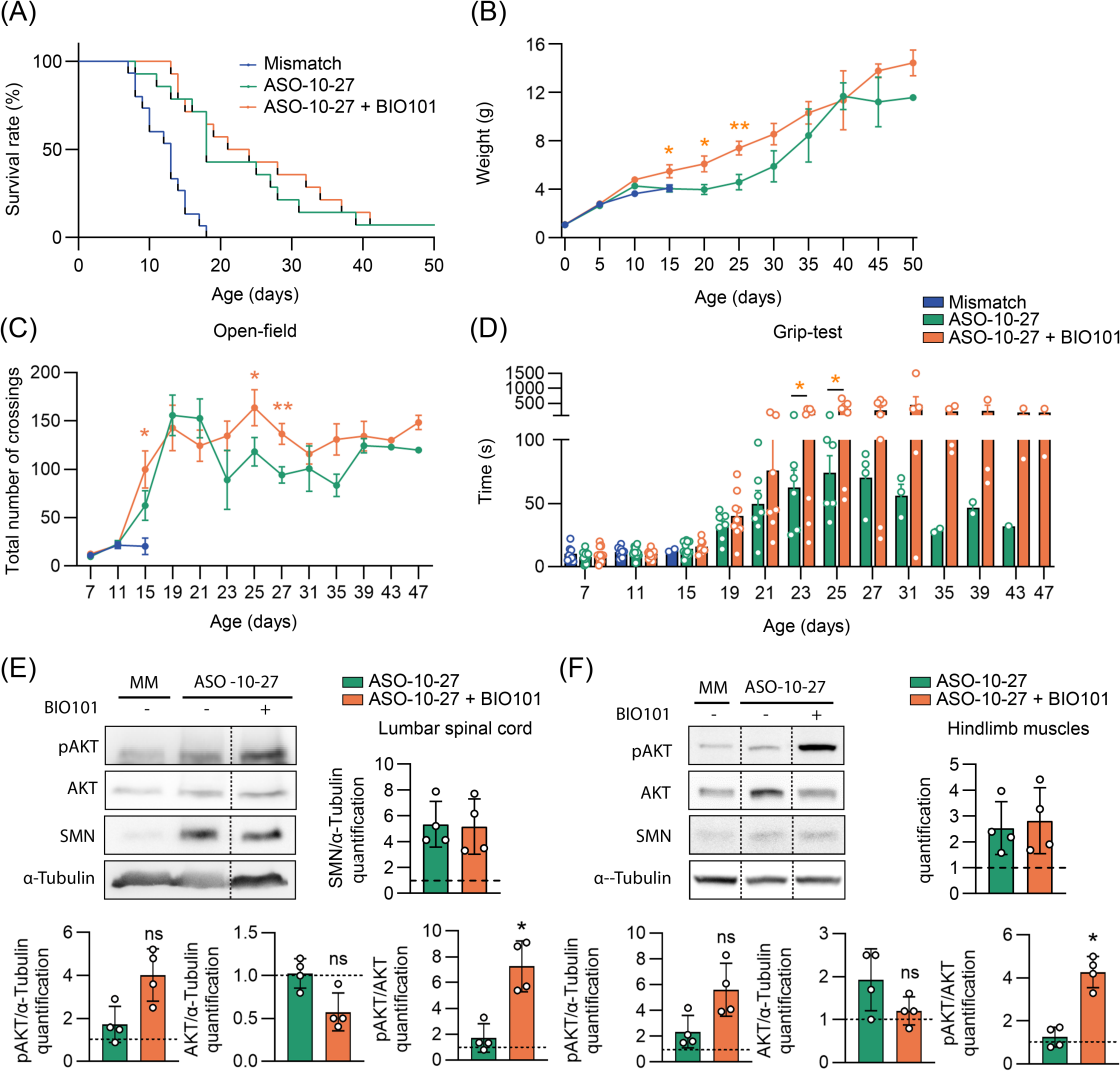
Effects of BIO101 in combination with ASO‐10‐27 on severe Taiwanese SMA mouse phenotype. (A) Survival curves of severe SMA mice treated with the ASO‐mismatch (*n* = 15 mice, median survival of 13 days), the ASO‐10‐27 (*n* = 14 mice, median survival of 18 days), or the ASO‐10‐27 combined with BIO101 (*n* = 14 mice, median survival of 22.5 days). (B, C and D) Weight curves (B), and quantification of the maximal time of hind‐limb suspension score (grip‐test, muscle fatigue) (C), and of the total number of squares crossed (open‐field, moving capacity) (D) of severe SMA mice treated with the ASO‐mismatch (*n* = 15 mice), the ASO‐10‐27 (*n* = 16 mice) or the ASO‐10‐27 combined with BIO101 (*n* = 15 mice). (E and F) Western Blot analysis and quantification of phosphorylated form of AKT (pAKT), total form of AKT (AKT) and SMN expression in the lumbar spinal cord (E) and in a pool of hindlimb *tibialis*, *plantaris* and *soleus* muscles (F) of severe SMA mice treated with the ASO‐mismatch, the ASO‐10‐27 or the ASO‐10‐27 combined with BIO101 (*n* = 2 ASO‐mismatch‐treated mice; *n* = 4 mice treated with ASO‐10‐27 ± BIO101; lanes were run on the same gel but were noncontiguous). (B–D) Data are represented as mean ± SEM while (E and F) data are represented as mean ± SD with *p < 0.05 and **p < 0.01 for a comparison between the two indicated groups or with vehicle‐treated control mice by (A) log‐rank test or (B, C and D) unpaired *t*‐test at each age or (E and F) unpaired nonparametric Mann–Whitney test.

Our results showed that BIO101 alone can positively affect the entire motor unit in severe SMA mice leading to specific motor function benefits whatever the age and the severity, and which is also relevant on SMA‐patient cells in an SMN‐independent manner. This led us to consider BIO101 as a potent combinatorial treatment with SMN‐based therapies known for their marked survival benefits with persistent muscular defects [11, S6]. The combination of a single intracerebroventricular (ICV) injection of 8 μg of ASO‐10‐27 at birth with the daily oral administration of BIO101 did not affect the survival gain observed in ASO‐10‐27‐treated severe SMA mice (log‐rank ***p = 0.0002; +38% in median survival) compared with mismatch‐treated severe SMA mice (median survival of 13 days; Figure 6A). A moderate weight gain was observed between P15 and P25 in co‐treated severe SMA mice compared with severe SMA mice treated with ASO‐10‐27 alone (Figure 6B). However, for moving capacities, we observed a significant increase in the total number of crossings at P15 (Figure 6C) followed by a higher plateau beyond P19 for the co‐treated severe SMA mice (Figure 6C). Most importantly, with the grip‐test, we noted a marked and significant increase of the time of suspension at P23 and P25 for co‐treated severe SMA mice compared with severe SMA mice treated with ASO‐10‐27 alone (Figure 6D), with an impressive maintenance in performance until 47 post‐natal days (Figure 6D). Thus, BIO101 specifically improves resistance to muscular fatigue in combination with SMN‐based therapy. Finally, despite the increase of SMN protein induced by ASO‐10‐27 injection and the proper triggering of the AKT intracellular pathway (Figure 6E,F), BIO101 treatment failed in increasing SMN protein expression at P10.5 in both spinal cord (Figure 6E) and hind limb muscles (Figure 6F) of co‐treated severe SMA mice compared with severe SMA mice treated with ASO‐10‐27 alone. These results highlight the promising ability of BIO101 to benefit muscular function in combination with ASO‐10‐27.

## 4 Discussion

The advances in SMN‐based therapy for SMA have completely changed disease progression, with the emergence of new symptoms, leading to consider complementary treatments targeting the remaining alteration of neuromuscular function and/or the non‐responding patients. We demonstrated that a daily oral administration of BIO101 can specifically improve muscular function in severe (infanthood) and mild (adulthood) Taiwanese SMA mouse models. The effects on severe SMA mice occurred through preferential protection of all the components of fast motor units. We demonstrated that BIO101 can also positively impact human myoblasts from type II SMA patients, favouring the differentiation, as previously demonstrated in mice or human control myoblasts. Importantly, all BIO101‐induced benefits are independent of SMN expression. Thus, when combined with the SMN‐based therapy ASO‐10‐27, BIO101 potentializes motor function improvement, highlighting the therapeutic potential of BIO101 for all severities of SMA.

Considering the crucial role of muscle function on the quality of life of SMA patients, the most striking result of our study relies on the ability of BIO101 to improve muscle performance in Taiwanese SMA mouse models (Figures 1E,F and 6D), administered alone or in combination with the ASO‐10‐27, whatever the considered severity and age. This specific functional effect of BIO101, without relevant effects on the survival of severe SMA mice, could rely on the limitation of muscular atrophy and the protection of lumbar motor neurons through direct or indirect effects. Indeed, the acceleration of the maturation of the neuromuscular structures could have an effect on cross‐section area of myofibers which both are known to favour muscular performance by enhancing muscle force and functional effectiveness [27, S23]. Those gains improve the ability of mice to move freely (open‐field) and to sustain longer muscular contraction for equivalent weight (grip‐test) due to lower involvement regarding maximal force, both parameters of muscular function with important clinical relevance for SMA patients. In addition, the impact of muscle state on motor neuron function and survival in SMA is well known [28]. Thus, by improving muscle phenotype in severe SMA mice, BIO101 could indirectly limit motor neuron defects through specific myokines release and retrograde factor production, both known to protect motor neurons even in SMA [29]. Ultimately, this muscle‐induced improvement of motor neuron state can positively loop to enhance in turn neuromuscular activity and therefore limit muscle atrophy [22, 30]. Moreover, BIO101 improved spinal cord vascularization, which could also participate in neuroprotection by favouring nutrient delivery and metabolism function [31].

We noted a poor amount of motor neuron degeneration, affecting preferentially the lateral subpopulation, a specific phenotype for our Taiwanese severe SMA mouse model when compared with previous studies on the same model [22, 32] and contrasting with a recent study showing no degeneration in this model [33]. This discrepancy confirms the constant evolution of the mouse model of SMA over time and generations, reinforcing the idea that SMA is a systemic disease leading to the alteration of the neuromuscular function through metabolic defects and synaptopathy. By its multi‐tissular effects, BIO101 seems to match perfectly to disease specificities and heterogeneity.

The BIO101‐induced limitation of muscular atrophy in severe SMA mice (Figure 4A) could be linked to the BIO101/MAS receptor intracellular signalling activation favouring an increase of proteosynthesis through the regulation of AKT/mTOR pathway and/or to the partial inhibition of myostatin gene expression [17, S15], already demonstrate as beneficial on animal models of SMA [34, 35]. Moreover, the ASO‐10‐27 treatment [34] is known to improve myostatin expression which could potentialize the effects of its inhibition through BIO101 treatment and explain the more pronounced functional benefits when both treatments are combined. However, the exact mechanisms by which BIO101 treatment exerts its effects need further investigation.

Another major aspect of BIO101 effects relies on the highest benefits observed in the fast‐twitch *tibialis* and *plantaris* muscles compared with the slow‐twitch *soleus* muscle, (Figures 3 and 4). These specific effects could be explained by muscle‐specific intracellular adaptations regarding their types, and notably to the Ca^2+^ homeostasis known to be highly altered in SMA [32, 36] and differentially regulated in slow‐ and fast‐twitch muscles [37, S26]. We know that MAS receptor allows transient Ca^2+^ release from the endoplasmic reticulum [S15]. Thus, imposing an equivalent influx of Ca^2+^ in a slow‐ versus fast‐twitch muscle with BIO101 treatment may not result in the same intracellular adaptations, notably through Ca2+‐sensitive signalling pathways like CAMKII, known to be altered in SMA [38]. Interestingly, the BIO101 effects on muscular vascularization also present this fast‐twitch preference, which could partly explain muscular improvement through metabolic benefits.

In addition, BIO101 seems to modulate the entire motor unit fate during development in favour of fast/intermediate motor unit in control and severe SMA mice, with a change in the proportion of MN+ ERRβ+ and MN+ MMP9+ (Figure S1E; Figure 2D,E), associated with a faster muscle typology with BIO101 treatment (Figure S3E; Figure 4D). This effect is also supported by the improvement of anaerobic performance of type II SMA myotubes (Figure 5C,D). This BIO101‐induced adaptation pushes energetic capacities and lowers the system requirements in mitochondrial function and vascularization, making it a drug candidate well adapted to known SMA alterations and condition [39]. Extending the study to SMA Type I and Type III myoblasts will also provide critical insights into different disease severity and potential specific adaptations.

Interestingly, all the beneficial effects of BIO101 are independent of SMN protein expression (Figures 4F, 5E, 6E and 6F), which reinforces its potent for combinatorial treatment and for mild and adult forms of the disease.

Finally, despite clear evidence of multi‐tissular and multi‐level benefits of BIO101 leading to muscular function improvement, the treatment presented negligible effects on survival in severe SMA mice, as monotherapy and in combination with ASO10‐27, when compared with other approaches like physical exercise, pharmacological activation of the NMDA receptor or genetic invalidation of the IGF‐1 receptor [22, 30, 38]. This peculiarity could be explained by a limited improvement in cardiac function [40, S27] and/or respiratory muscle function. However, even if the effects of BIO101 on cardiac tissue have not been investigated in this study and deserve further analysis, the molecule 20E, and by extension BIO101, is known to improve cardio‐vascular as well as respiratory function, even in disease context [S17, S28]. Thus, these contrasting results could rely either on a specific tissular and cellular status of SMA cardio‐respiratory system, which could limit the effect of BIO101, or on the cellular‐specific effect of BIO101, which can evolve along maturation and age, being more pronounced on cardio‐respiratory system at adulthood. This peculiarity needs to be extensively explored and described with specific research programs. However, this limitation observed on severe mouse model has a completely different interest for SMA patient clinical care. Patients can benefit from adapted clinical care with respiratory and nutritional aids, while motor function remains orphan of specific care, for patients treated or not with SMN‐based therapies.

To conclude, BIO101 appears as a new promising therapeutic option for SMA patients in combination with SMN‐dependent therapies such as ASO therapy, or as monotherapy for milder forms regarding its safety, as shown in clinical trials in adults or in non‐clinical studies in juvenile animals, and oral administration. An optimization of the BIO101 administration frequency could be necessary to enhance the beneficial effect of the molecule in SMA patients but its capacity to mediate different pharmacological effects according to the cellular context and to induce benefits on a wide range of severities and ages, makes it well‐adapted to the clinical heterogeneity of SMA.

## Conflicts of Interest

C.Be., S.C., P.N.H., C.Ba., R.L., S.V., P.D. and M.L. are, or were, employees of Biophytis and own equity in the company. C.Be., R.L., S.V., P.D., M.L., F.C. and O.B. are inventors or contributors on patent(s) concerning the use of BIO101 in SMA. They declare, however, that the company's potential commercial interests had no impact on the conduct of this research work.

## Supporting information


**Figure S1:** Effects of BIO101 on spinal cord in control mice. Quantification of the total number of ChAT+ cells depending on their localization (medial and lateral) (A) and of the distribution of their soma area (B) per 50‐μm‐thick slice of lumbar spinal cord of vehicle‐ compared with BIO101‐treated control mice at P10.5 (*n* = 5 mice in each group). (C) Quantification of the total number ERRβ+ ChAT+ (*n* = 7 BIO101‐treated SMA‐like mice, *n* = 6 in other groups) cells over total ChAT+ cells, in 50‐μm‐thick slice of lumbar spinal cord of vehicle‐treated control mice compared with vehicle‐ or BIO101‐treated SMA‐like mice at P10.5. (D and E) Quantification of the proportion of double positives MMP9‐ChAT (D) or ERRβ‐ChAT (E) cells over total ChAT+ cells in 50‐μm‐thick slice of lumbar spinal cord of vehicle‐ (*n* = 5 for the double positives MMP9‐ChAT; *n* = 6 for the double positives ERRβ‐ChAT) compared with BIO101‐treated control mice (*n* = 6) at P10.5. (F) Quantitative analysis of the capillary density in the ventral horn of lumbar spinal cord of vehicle‐ compared with BIO101‐treated control mice at P10.5 (*n* = 4 mice in each group). Data are represented as mean ± SD with *p < 0.05 for a comparison between the two indicated groups by (A, C, D, E and F) unpaired nonparametric Mann–Whitney tests or (B) two‐way ANOVA with a Sidak post hoc tests.

**Figure S2:** Effects of BIO101 on neuromuscular junction and muscle maturation in control mice. (A) Quantitative analyses of the percentage of uniform and perforated NMJ over total NMJ in the *tibialis*, *plantaris* and *soleus* muscles of vehicle‐ compared with BIO101‐treated control mice at P10.5 (*n* = 5 mice in each group). (B) Quantitative analysis of the percentage of myofibers expressing embryonic Myosin Heavy Chain isoform over total number of myofibers in the *tibialis*, *plantaris* and *soleus* muscles of vehicle‐ compared with BIO101‐treated control mice at P10.5 (*n* = 5 mice in each group). Data are represented as mean ± SD with *p < 0.05 for a comparison between the two indicated groups by (A) Kruskal–Wallis test with a Sidak post hoc tests or (B) unpaired nonparametric Mann–Whitney tests.

**Figure S3:** Effects of BIO101 on muscle phenotype in control mice. (A) Images of haematoxylin–eosin staining on *tibialis* muscles of vehicle‐treated control mice compared with vehicle‐ or BIO101‐treated SMA mice at P10.5 (scale bar: 25 μm) (B–D) Quantitative analysis of the mean cross‐sectional area of fibres (B), their standard deviation (C) and the total number of fibres (D) in the *tibialis*, *plantaris* and *soleus* muscles of vehicle‐ compared with BIO101‐treated control mice at P10.5 (*n* = 5 mice in each group). (E) Quantitative analysis of the percentage of myofibers expressing adult type 1, type 2A and type 2X/2B Myosin Heavy Chain isoforms over total number of myofibers in the *tibialis*, *plantaris* and *soleus* muscles of vehicle‐ (CV) compared with BIO101‐treated (CB) control mice at P10.5 (*n* = 5 mice in each group). (F) Quantitative analysis of the number of vessels per muscle fibre in the *tibialis*, *plantaris* and *soleus* muscle of vehicle‐ compared with BIO101‐treated control mice at P10.5 (*n* = 5 mice in each group). (G and H) Quantification of the relative mRNA levels of Mas in the *tibialis*, *plantaris* and *soleus* muscles of vehicle‐treated control mice (*n* = 5 in each group) (G) and in vehicle‐treated control mice compared with BIO101‐treated control mice and vehicle‐ or BIO101‐treated SMA‐like mice at P10.5 for the *tibialis*, *plantaris* and *soleus* muscles (*n* = 5 mice in each group) (H). Data are represented as mean ± SD with *p < 0.05 and **p < 0.01 for a comparison between the two indicated groups by (B, C, D and F) unpaired nonparametric Mann–Whitney tests or (E) two‐way ANOVA with a Sidak post hoc tests or (G and H) Kruskal–Wallis test with a Sidak post hoc tests.

**Figure S4:** Effects of BIO101 on SMA patient‐derived myoblast differentiation and metabolism. (A) Quantification of the relative mRNA levels of MyoD and Myogenin in myotubes derived from type 2 SMA patient treated during 2 (D2) and 6 (D6) days of differentiation with BIO101 (BIO) ± A779 (BIO + A779 or A779) compared with untreated (UT) myotubes (*n* = 3). (B and C) OCR Mito Stress test Seahorse profiles (B) and quantification of metabolic parameters (C) (BR, basal respiration; PL, proton leak; ATP, ATP production; MR, mitochondrial respiration; SRC, spare respiratory capacity; NMOC, non‐mitochondrial oxygen consumption), in myotubes derived from type 2 SMA patient treated during 2 days of differentiation with BIO101 ± A779 (*n* = 3). Data are represented as mean ± SD with *p < 0.05 for a comparison with UT condition (A) Kruskal–Wallis test with a Sidak post hoc tests.

**Table S1:** Supplementary Information.
**Table S2:** Supplementary Information.
**Table S3:** Supplementary Information.
**Table S4:** Supplementary Information.
**Table S5:** Supplementary Information.
**Table S6:** Supplementary Information.
