# Supplementary figures and images for: Drug Candidate BIO101 for Spinal Muscular Atrophy as Monotherapy or Combined With the Antisense Oligonucleotide ASO‐10‐27
Source: J Cachexia Sarcopenia Muscle. 2025 Oct 23;16(5):e70104. doi: 10.1002/jcsm.70104 (PMC12547075; doi:10.1002/jcsm.70104)

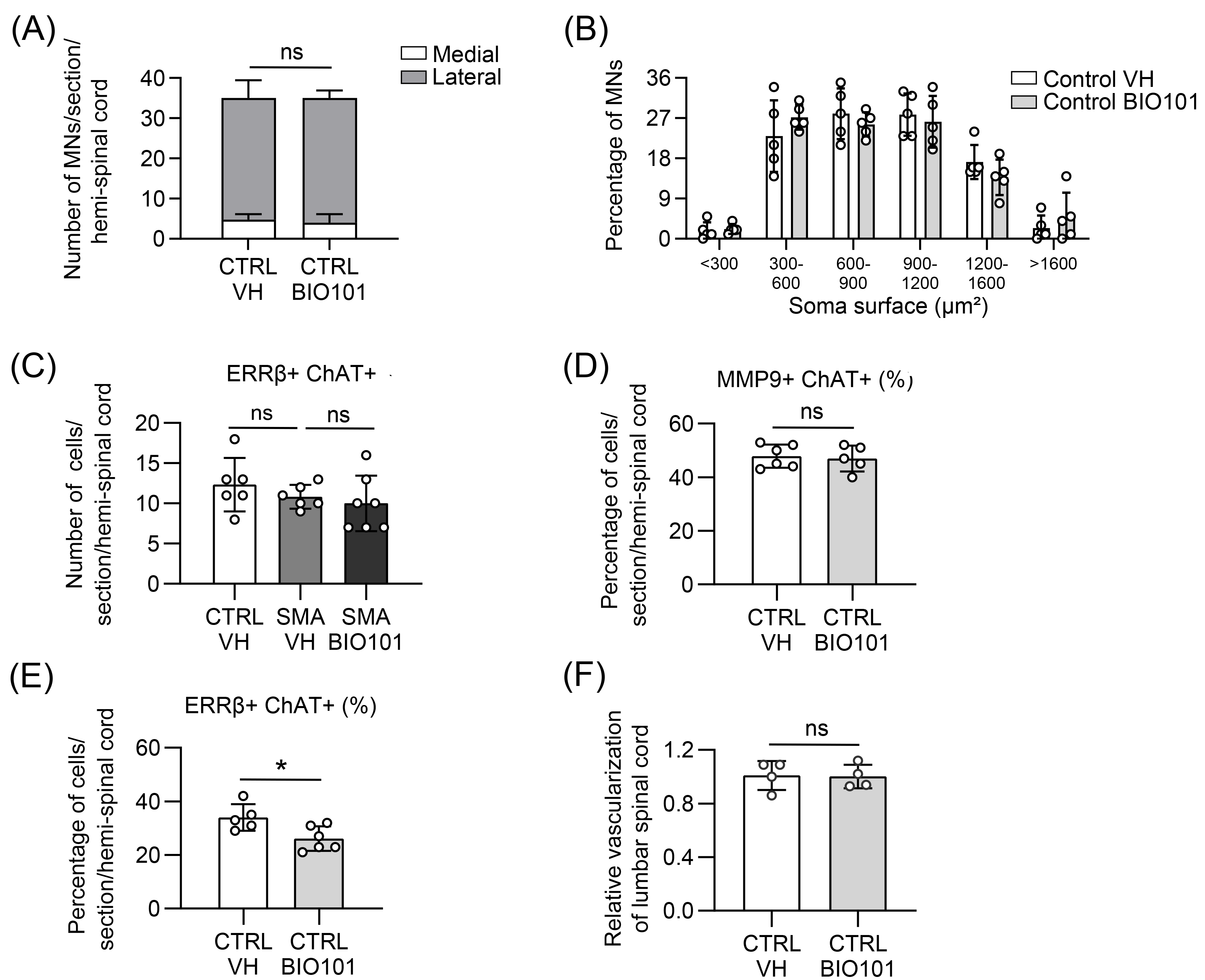

Supplement: Supplementary file 1 — Figure S1: Effects of BIO101 on spinal cord in control mice. Quantification of the total number of ChAT+ cells depending on their localization (medial and lateral) (A) and of the distribution of their soma area (B) per 50‐μm‐thick slice of lumbar spinal cord of vehicle‐ compared with BIO101‐treated control mice at P10.5 (n = 5 mice in each group). (C) Quantification of the total number ERRβ+ ChAT+ (n = 7 BIO101‐treated SMA‐like mice, n = 6 in other groups) cells over total ChAT+ cells, in 50‐μm‐thick slice of lumbar spinal cord of vehicle‐treated control mice compared with vehicle‐ or BIO101‐treated SMA‐like mice at P10.5. (D and E) Quantification of the proportion of double positives MMP9‐ChAT (D) or ERRβ‐ChAT (E) cells over total ChAT+ cells in 50‐μm‐thick slice of lumbar spinal cord of vehicle‐ (n = 5 for the double positives MMP9‐ChAT; n = 6 for the double positives ERRβ‐ChAT) compared with BIO101‐treated control mice (n = 6) at P10.5. (F) Quantitative analysis of the capillary density in the ventral horn of lumbar spinal cord of vehicle‐ compared with BIO101‐treated control mice at P10.5 (n = 4 mice in each group). Data are represented as mean ± SD with *p < 0.05 for a comparison between the two indicated groups by (A, C, D, E and F) unpaired nonparametric Mann–Whitney tests or (B) two‐way ANOVA with a Sidak post hoc tests. [file JCSM-16-e70104-s004.tif]

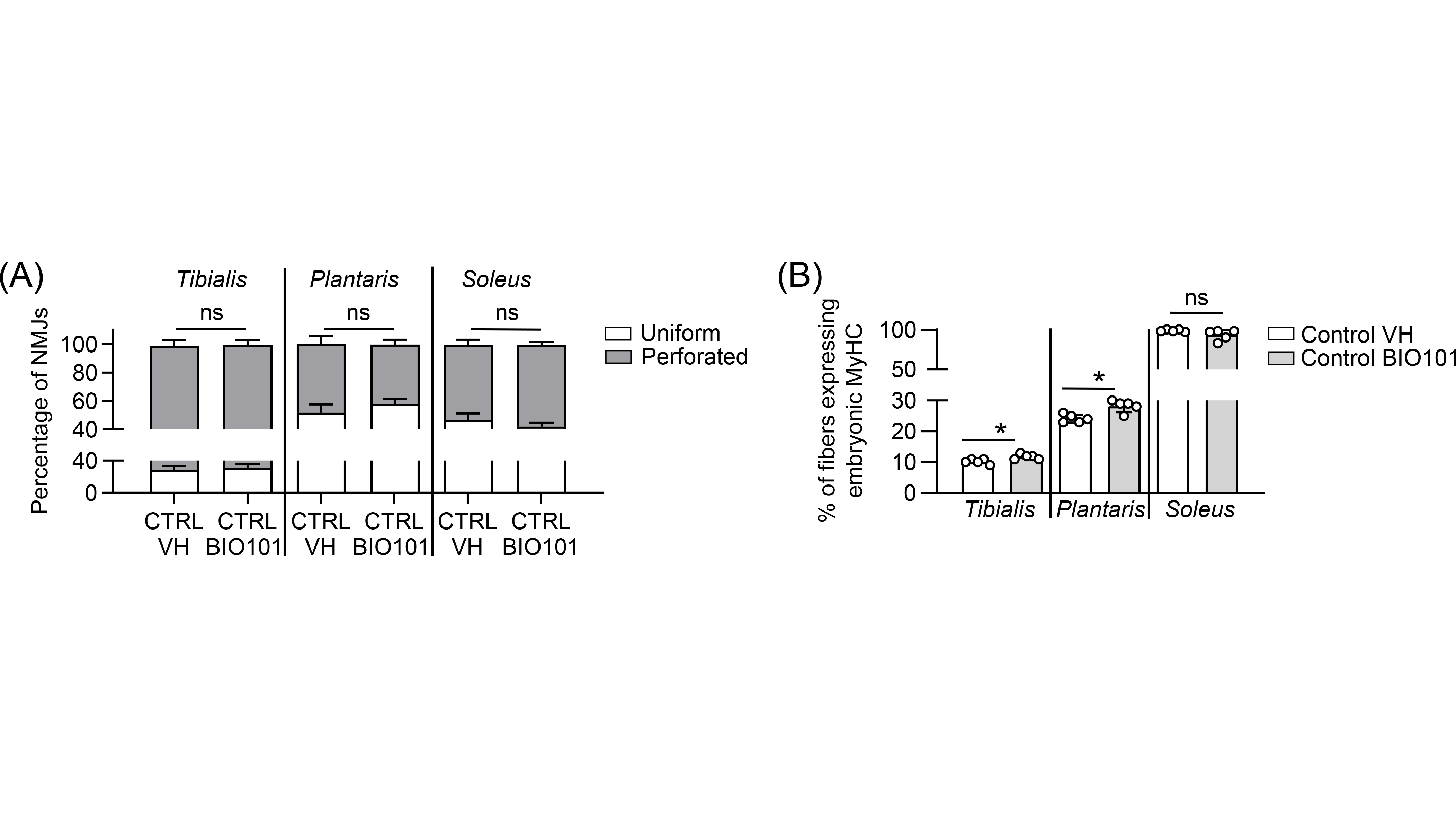

Supplement: Supplementary file 2 — Figure S2: Effects of BIO101 on neuromuscular junction and muscle maturation in control mice. (A) Quantitative analyses of the percentage of uniform and perforated NMJ over total NMJ in the tibialis, plantaris and soleus muscles of vehicle‐ compared with BIO101‐treated control mice at P10.5 (n = 5 mice in each group). (B) Quantitative analysis of the percentage of myofibers expressing embryonic Myosin Heavy Chain isoform over total number of myofibers in the tibialis, plantaris and soleus muscles of vehicle‐ compared with BIO101‐treated control mice at P10.5 (n = 5 mice in each group). Data are represented as mean ± SD with *p < 0.05 for a comparison between the two indicated groups by (A) Kruskal–Wallis test with a Sidak post hoc tests or (B) unpaired nonparametric Mann–Whitney tests. [file JCSM-16-e70104-s002.tif]

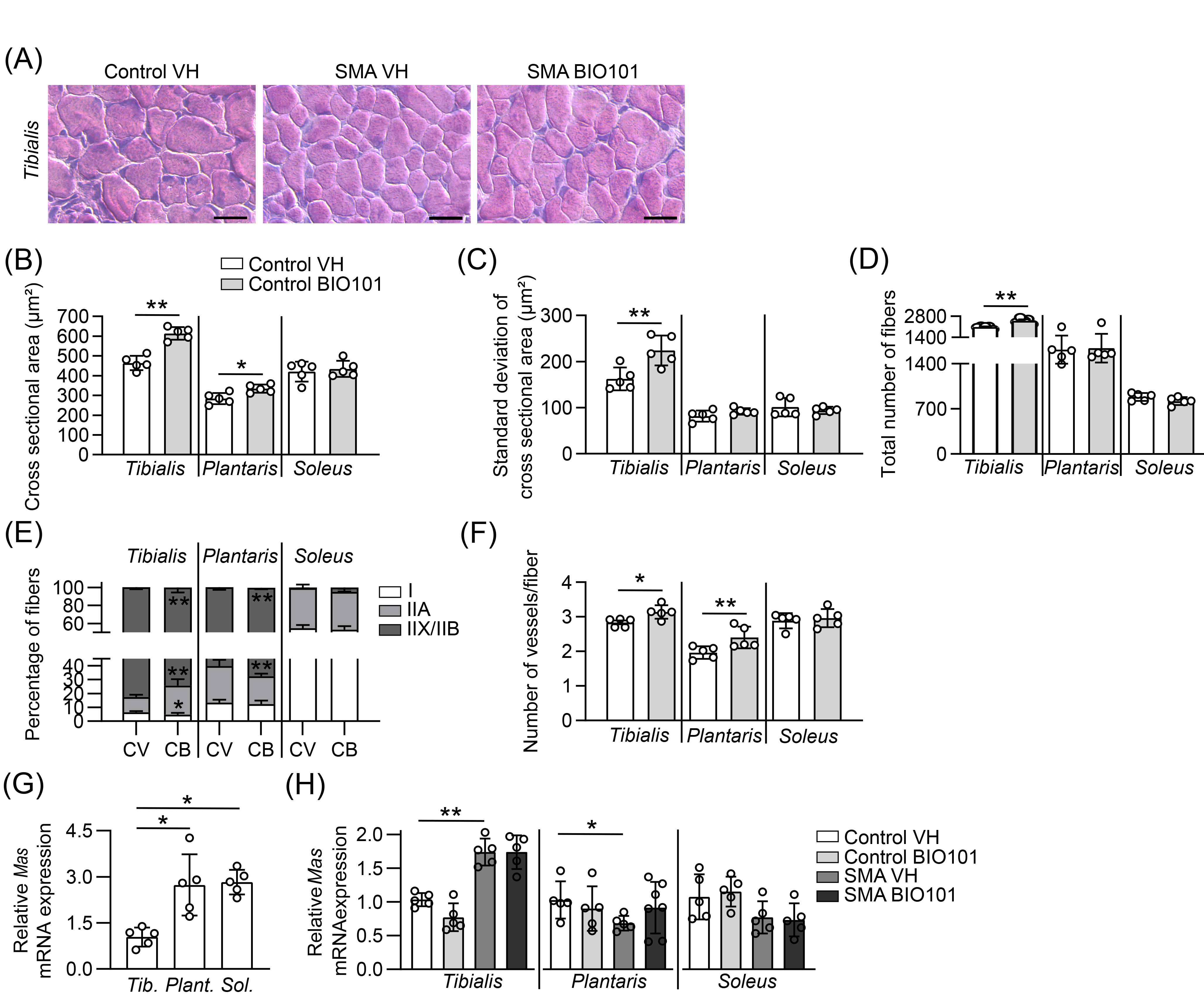

Supplement: Supplementary file 3 — Figure S3: Effects of BIO101 on muscle phenotype in control mice. (A) Images of haematoxylin–eosin staining on tibialis muscles of vehicle‐treated control mice compared with vehicle‐ or BIO101‐treated SMA mice at P10.5 (scale bar: 25 μm) (B–D) Quantitative analysis of the mean cross‐sectional area of fibres (B), their standard deviation (C) and the total number of fibres (D) in the tibialis, plantaris and soleus muscles of vehicle‐ compared with BIO101‐treated control mice at P10.5 (n = 5 mice in each group). (E) Quantitative analysis of the percentage of myofibers expressing adult type 1, type 2A and type 2X/2B Myosin Heavy Chain isoforms over total number of myofibers in the tibialis, plantaris and soleus muscles of vehicle‐ (CV) compared with BIO101‐treated (CB) control mice at P10.5 (n = 5 mice in each group). (F) Quantitative analysis of the number of vessels per muscle fibre in the tibialis, plantaris and soleus muscle of vehicle‐ compared with BIO101‐treated control mice at P10.5 (n = 5 mice in each group). (G and H) Quantification of the relative mRNA levels of Mas in the tibialis, plantaris and soleus muscles of vehicle‐treated control mice (n = 5 in each group) (G) and in vehicle‐treated control mice compared with BIO101‐treated control mice and vehicle‐ or BIO101‐treated SMA‐like mice at P10.5 for the tibialis, plantaris and soleus muscles (n = 5 mice in each group) (H). Data are represented as mean ± SD with *p < 0.05 and **p < 0.01 for a comparison between the two indicated groups by (B, C, D and F) unpaired nonparametric Mann–Whitney tests or (E) two‐way ANOVA with a Sidak post hoc tests or (G and H) Kruskal–Wallis test with a Sidak post hoc tests. [file JCSM-16-e70104-s003.tif]

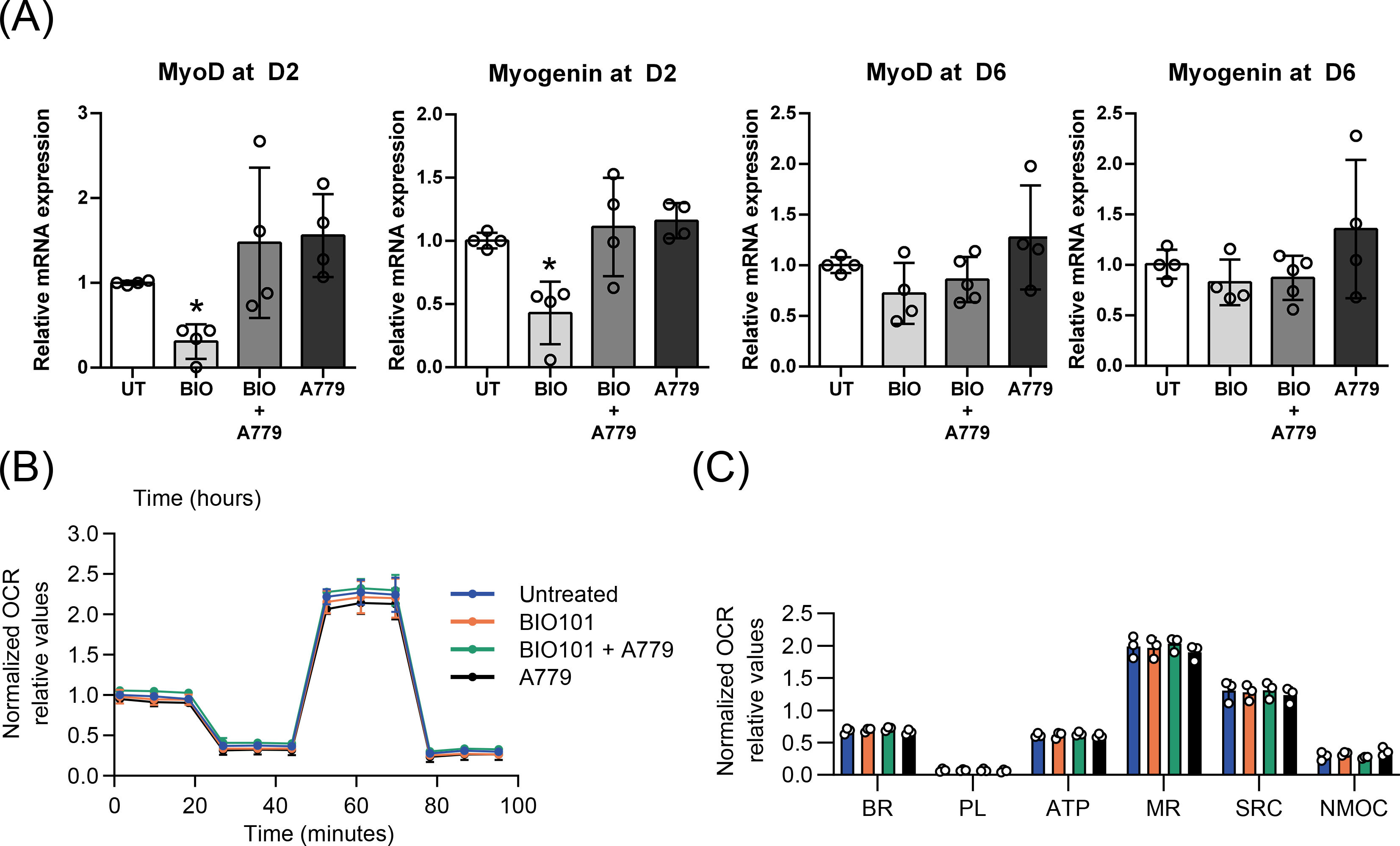

Supplement: Supplementary file 4 — Figure S4: Effects of BIO101 on SMA patient‐derived myoblast differentiation and metabolism. (A) Quantification of the relative mRNA levels of MyoD and Myogenin in myotubes derived from type 2 SMA patient treated during 2 (D2) and 6 (D6) days of differentiation with BIO101 (BIO) ± A779 (BIO + A779 or A779) compared with untreated (UT) myotubes (n = 3). (B and C) OCR Mito Stress test Seahorse profiles (B) and quantification of metabolic parameters (C) (BR, basal respiration; PL, proton leak; ATP, ATP production; MR, mitochondrial respiration; SRC, spare respiratory capacity; NMOC, non‐mitochondrial oxygen consumption), in myotubes derived from type 2 SMA patient treated during 2 days of differentiation with BIO101 ± A779 (n = 3). Data are represented as mean ± SD with *p < 0.05 for a comparison with UT condition (A) Kruskal–Wallis test with a Sidak post hoc tests. [file JCSM-16-e70104-s001.tif]
