# Supplementary material for: Drug Candidate BIO101 for Spinal Muscular Atrophy as Monotherapy or Combined With the Antisense Oligonucleotide ASO‐10‐27
Source: J Cachexia Sarcopenia Muscle. 2025 Oct 23;16(5):e70104. doi: 10.1002/jcsm.70104 (PMC12547075; doi:10.1002/jcsm.70104)
Supplement: Supplementary file 5 — Table S1: Supplementary Information. Table S2: Supplementary Information. Table S3: Supplementary Information. Table S4: Supplementary Information. Table S5: Supplementary Information. Table S6: Supplementary Information. [file JCSM-16-e70104-s005.pdf]

Supplementary Methods pages 2-5

Mice and treatments page 2

Motor behavior assays page 2

RNA extraction and RT-qPCR analysis page 3

High-Performance Liquid Chromatography (HPLC) page 3

Protein extraction and western blot analysis pages 4

Image acquisition and measurements page 4

Metabolic assay page 5

Supplementary Tables pages 6-9

Table S1 page 6

Table S2 page 7

Table S3 page 8

Table S4 page 8

Table S5 page 8

Table S6 page 9

Supplementary Figures pages10-13

Figure S1 page 9

Figure S2 page 11

Figure S3 page 12

Figure S4 page 13

Supplementary references pages 14-15

Supplementary Methods

Mice and treatments

Survival of severe Taiwanese SMA model mice (*Smn*$^{\Delta 7/\Delta 7}$; tg*SMN2*$^{0/+}$, FVB.Cg-Smn1t^{m1Hung} Tg(SMN2)2Hung/J, strain #005058) were determined when animals fail to stand on their paws in less than 10 seconds after being placed on their back.

From 6 to 9 months of age, mild Taiwanese SMA model mice (*Smn*$^{\Delta 7/\Delta 7}$; tg*SMN2*$^{+/+}$, FVB.Cg-Smn1t^{m1Hung} Tg(SMN2)2Hung/J, strain #005058) were individually housed and received either only water in drink feeder or 50mg/kg/day of BIO101 directly diluted in the water for a consumption of 4g of water per day. Drink feeder was weighted every day to ensure proper consumption and BIO101 solution treatment was renewed every two days.

Motor behavior assays.

Grip test. The time during which the mice were able to sustain their own weight holding onto a thin metal rail suspended at either 20 cm (pups from P0 to P60) or 50 cm (adult mice) from a table was recorded. Only the maximum value of five successive trials was recorded with at least a 1 min resting period between each try, according to the Treat-NMD guidelines (SOP (ID) Number DMD_M.2.1.004).

Open-field test. Each mouse was placed in the center of the squared open-field box and allowed to move freely for 5 min. The total number of squares crossed was scored manually by the experimenter. All the tests were made blind, the treatment assignment being unknown to the observers.

RNA extraction and RT-qPCR analysis

Total RNA was extracted from tissue homogenates using Trizol Reagent (15596018, Thermo Fisher Scientific) according to the manufacturer's recommendations, followed by a TURBO DNA-free™ Kit treatment (01050331, Invitrogen) and a cDNA synthesis from 1 µg RNA using ImProm-II™ Reverse Transcriptase (A3802, Promega). Quantitative real-time PCR was performed in triplicate with diluted cDNA using SYBR Green ROX Mix (01094990, Thermo Fisher Scientific), which contains 100 nM of primers (Supplementary Table 4), with an ABIPrism 7000 system (ABgene). The normalized expression levels were calculated using to the $2^{-\Delta\Delta Ct}$ method to establish the relative expression ratio between experimental groups.

High-Performance Liquid Chromatography (HPLC)

Plasma samples were deproteinized by adding 4 volumes of methanol containing internal standard (Cyasterone). Standard and quality control samples were prepared in control plasma and treated in the same conditions to obtain a calibration curve (10 to 10000ng/mL). At a column temperature at 35°C, samples were analyzed on a Fortis C18 (5µm, 2.1x50mm) column and sample injection column of 10µL was used. The mobile phase consisted of mixture of solvent A (0.1%HCOOH in Milli-Q water) and solvent B (0.1%HCOOH in acetonitrile). The gradient program for HPLC analysis started with 85% solvent A and 15% solvent B, increasing linearly to 55% solvent B at t=8minutes.All samples were kept at 8°C before analysis. In the MRM mode, 20-hydroxyecdysone (481.1→371.2), and internal standard, Cyasterone (521.1→485.5) were followed and quantified with calibration curve performed from standard and controls samples.

Protein extraction and western blot analysis

We dissolved cells and tissues in blending buffer (50 mM Tris, 150 mM NaCl, 0.1% SDS, 1% NP40, 10 mM NaF, 1X protease inhibitor cocktail (#05892791001, Sigma), 5mM sodium orthovanadate, 5mM β-glycerophosphate, 10mM sodium pyrophosphate, 0.5M EDTA) by mechanically stressing the samples for 6 rounds of 30s at 25Hz with a 10min rest on ice between each round. For electrophoresis, we used a 12.5% SDS-PAGE (Stacking gel: 0.1M Tris pH 6.8, 3.6% acrylamide/bis solution (29:1), 0.1% SDS, 0.06% ammonium persulfate, 0.2% tetramethylethylenediamine; Separating gel: 0.4M Tris pH 8.8, 12.5% acrylamide/bis solution (29:1), 0.1% SDS, 0.06% ammonium persulfate, 0.1% tetramethylethylenediamine). After transfer, equal loading of samples was checked by Ponceau dye staining of the transferred gels. After incubation in stripping buffer (0.7% β-mercaptoethanol, 2% SDS and 62.5mM Tris, pH 6.7) for 30 min at 55°C, blots were blocked again in TBS with 0,1% Tween-20 and 5% milk powder (1 h, RT) before incubation with a new primary antibody.

Image acquisition and measurements

For muscle histology and typology, fibers were counted manually for one section per muscle for each mouse. For the cross-section area analysis, 20% of total fibers was measured using 5 random areas across the section for one section per muscle for each mouse. **For the aplasia analysis, the absolute number of fibers per muscle was counted.** For typology analysis, the percentage for each MyHC has been expressed in percentage compared to the total number of fibers per muscle.

For muscle vascularization analysis, the number of vessels per fiber of 20% of fibers per section and per muscle was counted using 5 random areas across the section. For spinal cord vascularization analysis, images were converted to binary using Image J software (NIH). In the

resulting images, capillaries were assigned to black and spinal cord background to white. The ratio of black to total pixels was calculated and used to generate a value for capillary area in mm^2 per mm^2 of spinal cord as previously described [27].

For the NMJ analysis, 4 sections were analyzed for each muscle (around 150 NMJs per muscle). The maturation stage of each NMJ was determined according to their shape, known to reflect the distribution of acetylcholine receptors (α-bungarotoxin staining), as previously described[S29]. For spinal cord immunofluorescence, ventral horn motor neurons were manually counted on ≥ 8 sections per spinal cord and per mouse uniformly distributed throughout the lumbar region (from L1 to L5) for each mouse.

Metabolic assay.

OCR and ECAR were measured in XF media (non-buffered DMEM containing 25 mM glucose, 4 mM L-glutamine, and 1 mM sodium pyruvate) under basal conditions and in response to 1.5µM oligomycin, 5µM fluoro-carbonyl cyanide phenylhydrazone (FCCP) and 0.5µM rotenone + 0.5µM antimycin A with the XFe24 Analyzer (Agilent Technologies). For each experiment, 5 wells per condition were grouped based on their entry by the investigator into the Seahorse software "Group" column to produce a single overall metabolic profile. To account for plate-to-plate variation, the basal respiration measurement of the first replicate within the untreated group was baselined on 1 and all values were calculated relatively to this experiment. Replicates having OCR or ECAR measurement below zero were assumed to be in error and were excluded from calculations of metabolic profiles and parameters. The OCR:ECAR ratio at basal respiration for each group was reported as the mean of the ratios from measurements from 0 to 18 minutes. The OCR:ECAR ratio at maximal respiration for each group was reported as the ratio from the measurement with the highest OCR in the set of measurements from 53 to 70 minutes.

Table S1

	Muscle fibers maturation and typology	*Vascularization quantification*	*Neuromuscular junction maturation*	*Motor neuron counting and typology*
**Fixation**	N/A	10 min into cold methanol (-20°C)	N/A	N/A
**Blocking**	In PBS with 8% goat serum and 0.5% Tween-20, 30 min at room temperature	In PBS with 20% fetal bovine serum and 0.05% Tween-20, 30 min at room temperature	In PBS with 0,5% Triton X-100 3% bovine serum albumin and 5% goat serum, 4 h at room temperature	In TBS with 0.1M glycine for 1 h at room temperature, then in TBS with 0.5% Tween-20, 0.5% Triton X-100 and 8% donkey serum for 1 h at room temperature
**Primary antibodies**	In PBS with 8% goat serum and 0.5% Tween-20, 24 h at 4°C	In PBS with 0.05% Tween-20 and 1% FBS, 3 hours at room temperature	In PBS with 0,5% Triton X-100 3% bovine serum albumin and 5% goat serum, 2 days at 4°C	In TBS with 0.5% Tween-20, 0.5% Triton X-100 and 8% donkey serum, 2 days at 4°C
**Washing**	3 times for 10 min in PBS with 0.5% Tween-20	3 times for 10 min in PBS with 0.05% Tween-20	6 times for 30 min in PBS with 0.5% Triton X-100	3 times for 10 min in TBS with 0.5% Tween-20 and 0.5% Triton X-100
**Secondary antibodies**	In PBS with 0.5% Tween-20 and 4% goat serum, 1 h at room temperature	In PBS with 0.05% Tween-20 and 1% fetal bovine serum, 1 h at room temperature	Incubation with anti-rabbit Alexa Fluor 647 in blocking solution, 24 h at 4°C, then 6x30 min wash in PBS with 0.5% Triton X-100, and incubation with Alexa Fluor 555-conjugated-α-bungarotoxin in blocking solution, 1 h at room temperature	In TBS with 0.1% Tween-20, 0.5% Triton X-100 and 4% donkey serum, 1 h at room temperature
**Washing**	2 times for 10 min in PBS with 0.5% Tween-20 then once in PBS	2 times for 10 min in PBS with 0.05% Tween-20	2 times for 10 min in PBS with 0.5% Triton X-100 then once in PBS	3 times for 10 min in TBS with 0.5% Tween-20 and 0.5% Triton X-100
**Mounting**	In Fluoromount-G™ Mounting Medium (15586276, Invitrogen, Life Technologies)			

Table S2

<table>
<tr><th></th><th></th><th>Name</th><th>Reference</th><th>Supplier</th><th>Dilution</th></tr>
<tr><td rowspan="10">Muscle fibers maturation and typology</td><td rowspan="6">Primary antibodies</td><td>mouse anti-MYH3, IgG1</td><td>F1.652</td><td>DSHB</td><td>1:40</td></tr>
<tr><td>mouse anti-MYH7, IgG2b</td><td>BA-F8</td><td>DSHB</td><td>1:40</td></tr>
<tr><td>mouse anti-MYH2, IgG1</td><td>Sc-71</td><td>DSHB</td><td>1:200</td></tr>
<tr><td>mouse anti-MYH1, IgM</td><td>6H1</td><td>DSHB</td><td>1:40</td></tr>
<tr><td>mouse anti-MYH4, IgM</td><td>BF-F3</td><td>DSHB</td><td>1:200</td></tr>
<tr><td>rabbit anti-laminin</td><td>L9393</td><td>Sigma</td><td>1:750</td></tr>
<tr><td rowspan="4">Secondary antibodies</td><td>goat anti-mouse Alexa Fluor 350 anti-IgG2b</td><td>10042622</td><td>Thermo Fisher Scientific</td><td>1:500</td></tr>
<tr><td>goat anti-mouse Alexa Fluor 568 anti-IgG1</td><td>10483012</td><td>Thermo Fisher Scientific</td><td>1:1000</td></tr>
<tr><td>goat anti-mouse Alexa Fluor 647 anti-IgM</td><td>10368172</td><td>Thermo Fisher Scientific</td><td>1:1000</td></tr>
<tr><td>goat anti-rabbit Alexa Fluor 488 anti-IgG</td><td>10729174</td><td>Thermo Fisher Scientific</td><td>1:1000</td></tr>
<tr><td rowspan="4">Vascularization</td><td rowspan="2">Primary antibodies</td><td>rat anti-CD31</td><td>MA1-40074</td><td>Thermo Fisher Scientific</td><td>1:50</td></tr>
<tr><td>rabbit anti-laminin</td><td>L9393</td><td>Sigma</td><td>1:750</td></tr>
<tr><td rowspan="2">Secondary antibodies</td><td>goat anti-rat Cy3</td><td>112-166-003</td><td>Jackson ImmunoResearch</td><td>1:400</td></tr>
<tr><td>goat anti-rabbit Alexa Fluor 488</td><td>10729174</td><td>Thermo Fisher Scientific</td><td>1:1000</td></tr>
<tr><td rowspan="4">NMJ maturation</td><td rowspan="2">Primary antibodies</td><td>rabbit anti-neurofilament L (NF-L)</td><td>AB9568</td><td>Millipore</td><td>1:800</td></tr>
<tr><td>rabbit anti-synaptosomal-associated protein 25 (SNAP-25)</td><td>HPA001830</td><td>Sigma</td><td>1:200</td></tr>
<tr><td rowspan="2">Secondary antibodies</td><td>goat anti-rabbit Alexa Fluor 647</td><td>111-605-003</td><td>Jackson ImmunoResearch</td><td>1:400</td></tr>
<tr><td>Alexa Fluor 555-conjugated-α-bungarotoxin</td><td>B35451</td><td>Molecular probes</td><td>1:500</td></tr>
<tr><td rowspan="6">MN structure and typology</td><td rowspan="3">Primary antibodies</td><td>goat anti-Choline Acetylcholine Transferase (ChAT)</td><td>AB144P</td><td>Millipore</td><td>1:400</td></tr>
<tr><td>mouse anti-Estrogen-Related Receptor β (ERRβ)</td><td>PP-h6705-00 clone H705</td><td>R&D systems Europe</td><td>1:400</td></tr>
<tr><td>rabbit anti-Matrix MetalloPeptidase 9 (MMP9)</td><td>AB38898</td><td>Abcam</td><td>1:400</td></tr>
<tr><td rowspan="3">Secondary antibodies</td><td>donkey anti-goat Alexa Fluor Cy5</td><td>705-175-147</td><td>Jackson ImmunoResearch</td><td>1:400</td></tr>
<tr><td>donkey anti-mouse Alexa Fluor 488</td><td>715-545-150</td><td>Jackson ImmunoResearch</td><td>1:400</td></tr>
<tr><td>donkey anti-rabbit Alexa Fluor Cy3</td><td>711-165-152</td><td>Jackson ImmunoResearch</td><td>1:400</td></tr>
</table>

Table S3

Primary antibodies	rabbit anti-phospho-AKT (Ser473)	4058	Cell Signaling	1:1000
	rabbit anti-AKT	4691	Cell Signaling	1:1000
	mouse anti-SMN	610646	BD Transduction Laboratories	1:5000
	mouse anti-α-tubulin	T6074	Sigma-Aldrich	1:50 000
Secondary antibodies	horseradish peroxidase-conjugated goat anti-rabbit	111-035-003	Jackson ImmunoResearch	1:20 000
	horseradish peroxidase-conjugated goat anti-mouse	170-6516	Jackson ImmunoResearch	1:10 000

Table S4

Component	Concentration
**Growth medium (GM)**	
DMEM, high glucose, GlutaMAX^{TM} (61965, Thermo Fisher Scientific)	
heat-inactivated fetal bovine serum (FBS) (10500064, Gibco™)	20%
pyruvate (11360039, Gibco™, Thermo Fisher Scientific)	1%
penicilline-streptomycine (15140122, Gibco™, Thermo Fisher Scientific)	1%
ultroserG (15950-017, Sartorius, Germany)	2%
**Differenciation medium (DM)**	
DMEM, high glucose, GlutaMAX^{TM} (61965, Thermo Fisher Scientific)	
horse serum (26050088, Gibco™, Thermo Fisher Scientific)	0.5%
pyruvate (11360039, Gibco™, Thermo Fisher Scientific)	1%
penicilline-streptomycine (15140122, Gibco™, Thermo Fisher Scientific)	1%

Table S5

**Non mitochondrial respiration (nMitoR) = median (M_{10}, M_{11}, M_{12})**
**Proton leak (PL) = median (M_4, M_5, M_6) – nMitoR**
**Basal respiration (BR) = mean (M_1, M_2, M_3) - nMitoR**
**ATP production (ATP) = BR - PL**
**Maximal respiration (MR) = max (M7, M8, M9) - nMitoR**
**Spare respiratory capacity (SRC) = MR – BR**
**Where M is the set of 12 OCR measurements.**

Table S6

Primers	Sequence (5'->3')	Reference
Mas forward	TCATGTGTATTGACAGCGGAGAA	(72)
Mas reverse	CACTAACATGAGCGGAGTGAAGA	
**MyoD forward**	**CGAGCACTACAGCGGCGACT**	
**MyoD reverse**	**AGTCCATCATGCCGTCGGAGC**	
**Myogenin forward**	**CCAGGGGTGCCCAGCGAATG**	
**Myogenin reverse**	**TCAGCCGTGAGCAGATGATCCCC**	
RPL13 forward	AGGGGCAGGTTCTGGTATTG	(73)
RPL13 reverse	TGTTGATGCCTTCACAGCGT	
PPIA forward	GGCAAATGCTGGACCAAAC	
PPIA reverse	CATTCCTGGACCCAAAACG	
26S forward	AGAAGAAACAACGGTCGCGCCAAA	
26S reverse	GCGCAAGCAGGTCTGAATCGTG	

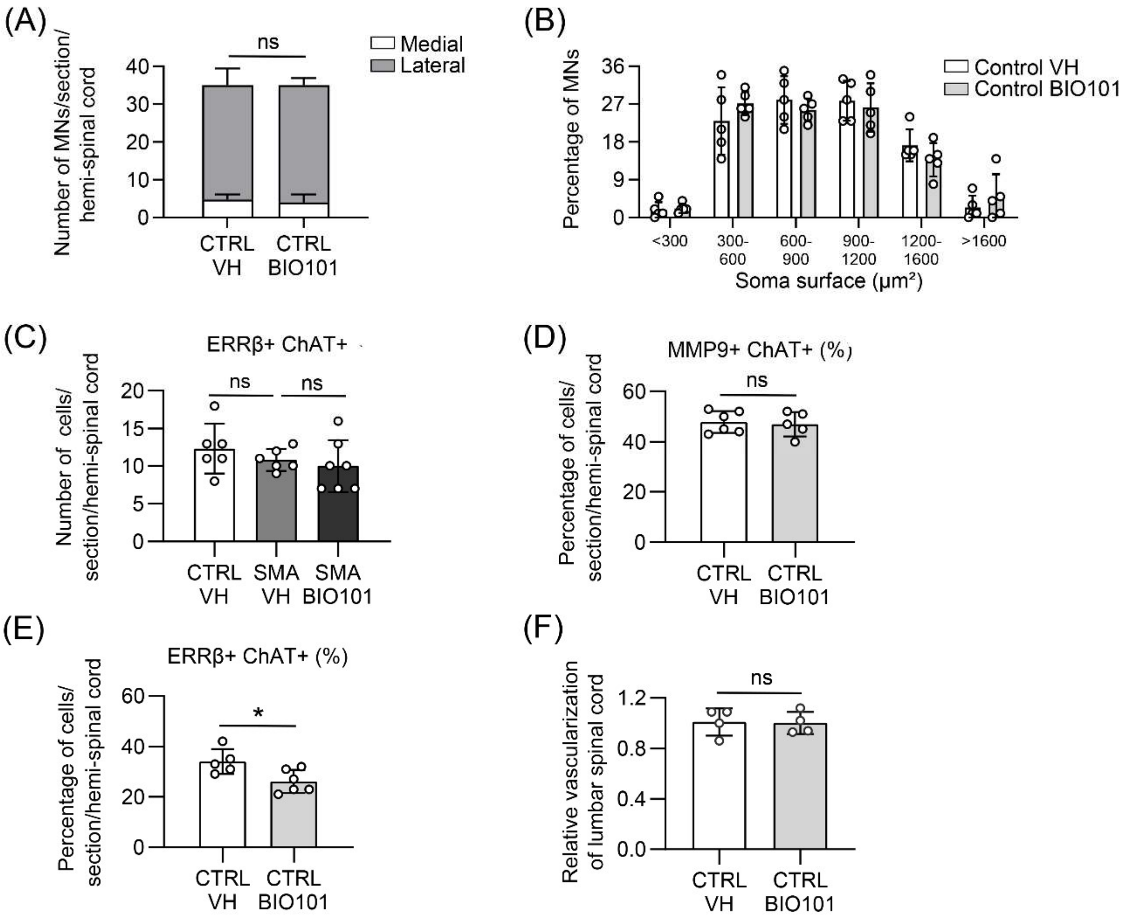

Figure S1: Effects of BIO101 on spinal cord in control mice. **(A and B)** Quantification of the total number of ChAT+ cells depending on their localization (medial and lateral) **(A)**, and of the distribution of their soma area **(B)** per 50-µm-thick slice of lumbar spinal cord of vehicle- compared to BIO101-treated control mice at P10.5 (n=5 mice in each group). **(C) Quantification of the total number ERRβ+ ChAT+ (n=7 BIO101-treated SMA-like mice, n=6 in other groups) cells over total ChAT+ cells, in 50-µm-thick slice of lumbar spinal cord of vehicle-treated control mice compared to vehicle- or BIO101-treated SMA-like mice at P10.5.** **(D and E)** Quantification the proportion of double positives MMP9-ChAT **(D)** or ERRβ-ChAT **(E)** cells over total ChAT+ cells in 50-µm-thick slice of lumbar spinal cord of vehicle- (n=5 for the double positives MMP9-ChAT; n=6 for the double positives ERRβ-ChAT) compared to BIO101-treated control mice (n=6) at P10.5. **(F)** Quantitative analysis of the capillary density in the ventral horn of lumbar spinal cord of vehicle- compared to BIO101-treated control mice at P10.5 (n=4 mice in each group). Data are represented as mean +/- SD with *p<0.05 for a comparison between the two indicated groups by **(A, C, D, E and F)** unpaired non-parametric Mann-Whitney tests or **(B)** two-way ANOVA with a Sidak *post-hoc* tests.

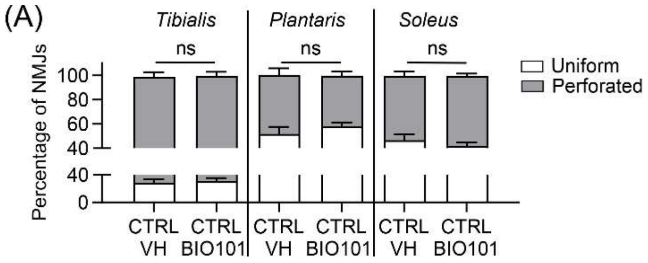

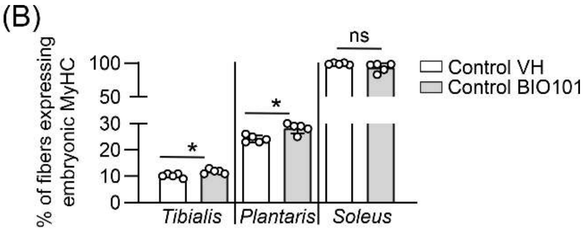

Figure S2: Effects of BIO101 **on neuromuscular junction and muscle maturation** in control mice. **(A)** Quantitative analyses of the percentage of uniform and perforated NMJ over total NMJ in the *tibialis*, *plantaris* and *soleus* muscles of vehicle- compared to BIO101-treated control mice at P10.5 (n=5 mice in each group). **(B)** Quantitative analysis of the percentage of myofibers expressing embryonic Myosin Heavy Chain isoform over total number of myofibers in the *tibialis*, *plantaris* and *soleus* muscles of vehicle- compared to BIO101-treated control mice at P10.5 (n=5 mice in each group). Data are represented as mean +/- SD with *p<0.05 for a comparison between the two indicated groups by **(A)** Kruskal-Wallis test with a Sidak *post-hoc* tests or **(B)** unpaired non-parametric Mann-Whitney tests.

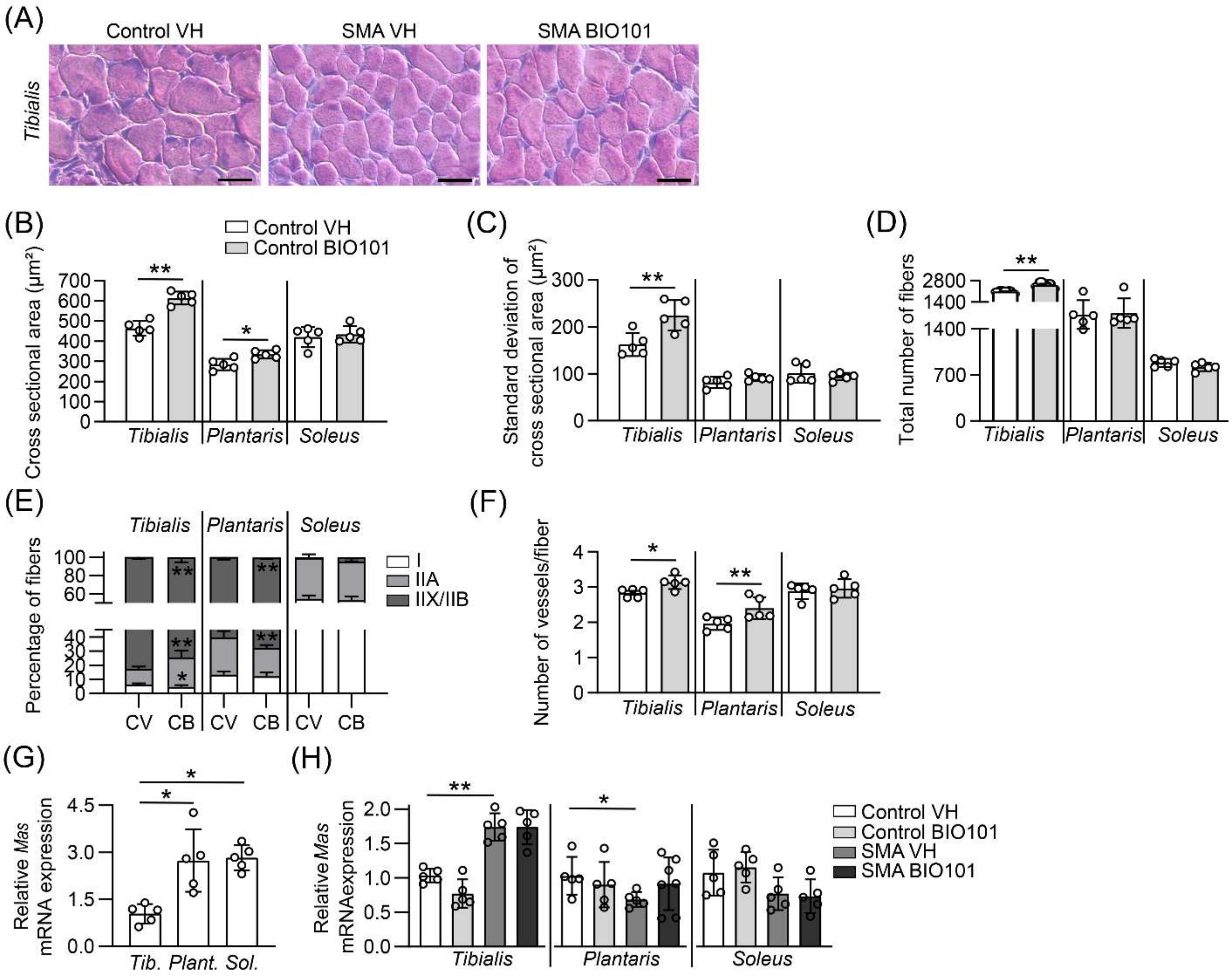

Figure S3: Effects of BIO101 on muscle phenotype in control mice. **(A) Images of hematoxylin-eosin staining on *tibialis* muscles of vehicle-treated control mice compared to vehicle- or BIO101-treated SMA mice at P10.5 (Scale bar: 25µm)** (**B-D**) Quantitative analysis of the mean cross-sectional area of fibers (**B**), their standard deviation (**C**) and the total number of fibers (**D**) in the *tibialis*, *plantaris* and *soleus* muscles of vehicle- compared to BIO101-treated control mice at P10.5 (n=5 mice in each group). (**E**) Quantitative analysis of the percentage of myofibers expressing adult type 1, type 2A and type 2X/2B Myosin Heavy Chain isoforms over total number of myofibers in the *tibialis*, *plantaris* and *soleus* muscles of vehicle- (CV) compared to BIO101-treated (CB) control mice at P10.5 (n=5 mice in each group). (**F**) Quantitative analysis of the number of vessels per muscle fiber in the *tibialis*, *plantaris* and *soleus* muscle of vehicle- compared to BIO101-treated control mice at P10.5 (n=5 mice in each group). (**G and H**) Quantification of the relative mRNA levels of *Mas* in the *tibialis*, *plantaris* and *soleus* muscles of vehicle-treated control mice (n=5 in each group) (**G**) and in vehicle-treated control mice compared to BIO101-treated control mice and vehicle- or BIO101-treated SMA-like mice at P10.5 for the *tibialis*, *plantaris* and *soleus* muscles (n=5 mice in each group) (**H**). Data are represented as mean +/- SD with *p<0.05, **p<0.01 for a comparison between the two indicated groups by (**B, C, D and F**) unpaired non-parametric Mann-Whitney tests or (**E**) two-way ANOVA with a Sidak *post-hoc* tests or (**G and H**) Kruskal-Wallis test with a Sidak *post-hoc* tests.

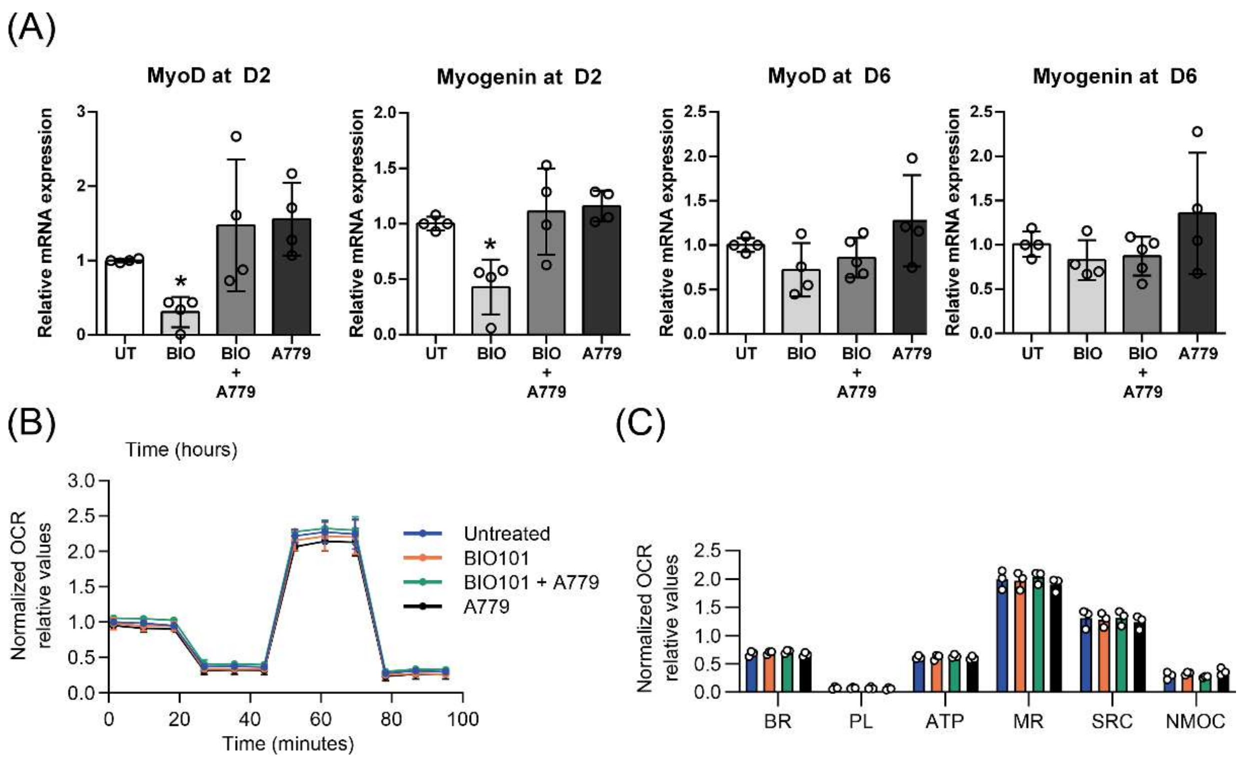

Figure S4: **Effects of BIO101 on SMA patient-derived myoblast differentiation and metabolism. (A) Quantification of the relative mRNA levels of *MyoD* and *Myogenin* in myotubes derived from type 2 SMA patient treated during 2 (D2) and 6 (D6) days of differentiation with BIO101 (BIO) +/- A779 (BIO+A779 or A779) compared to untreated (UT) myotubes (n=3). (B and C) OCR Mito Stress test Seahorse profiles (B) and quantification of metabolic parameters (C) (BR: Basal Respiration, PL: Proton Leak, ATP: ATP production, MR: Mitochondrial respiration, SRC: Spare Respiratory Capacity, NMOC: Non-Mitochondrial Oxygen Consumption), in myotubes derived from type 2 SMA patient treated during 2 days of differentiation with BIO101 +/- A779 (n=3). Data are represented as mean +/- SD with *p<0.05 for a comparison with UT condition (A) Kruskal-Wallis test with a Sidak *post-hoc* tests.**

Supplementary references

S1. Munsat TL, Davies KE. International SMA consortium meeting. (26-28 June 1992, Bonn, Germany). Neuromuscul Disord. 1992;2(5-6):423-8.

S2. Kashima T, Manley JL. A negative element in SMN2 exon 7 inhibits splicing in spinal muscular atrophy. Nat Genet. 2003;34(4):460-3.

S3. Cartegni L, Krainer AR. Disruption of an SF2/ASF-dependent exonic splicing enhancer in SMN2 causes spinal muscular atrophy in the absence of SMN1. Nat Genet. 2002;30(4):377-84.

S4. Mercuri E, Darras BT, Chiriboga CA, Day JW, Campbell C, Connolly AM, et al. Nusinersen versus Sham Control in Later-Onset Spinal Muscular Atrophy. N Engl J Med. 2018;378(7):625-35.

S5. Ramos DM, d'Ydewalle C, Gabbeta V, Dakka A, Klein SK, Norris DA, et al. Age-dependent SMN expression in disease-relevant tissue and implications for SMA treatment. J Clin Invest. 2019;129(11):4817-31.

S6. Arnold W, Severyn S, Zhao S, Kline D, Linsenmayer M, Kelly K, et al. Persistent neuromuscular junction transmission defects in adults with spinal muscular atrophy treated with nusinersen. BMJ Neurology Open. 2021;3:e000164.

S7. Feng Z, Ling KK, Zhao X, Zhou C, Karp G, Welch EM, et al. Pharmacologically induced mouse model of adult spinal muscular atrophy to evaluate effectiveness of therapeutics after disease onset. Hum Mol Genet. 2016;25(5):964-75.

S8. Muramatsu H, Kuramochi T, Katada H, Ueyama A, Ruike Y, Ohmine K, et al. Novel myostatin-specific antibody enhances muscle strength in muscle disease models. Sci Rep. 2021;11(1):2160.

S9. Liu M, Hammers DW, Barton ER, Sweeney HL. Activin Receptor Type IIB Inhibition Improves Muscle Phenotype and Function in a Mouse Model of Spinal Muscular Atrophy. PLoS One. 2016;11(11):e0166803.

S10. Gorelick-Feldman J, Maclean D, Ilic N, Poulev A, Lila MA, Cheng D, et al. Phytoecdysteroids increase protein synthesis in skeletal muscle cells. J Agric Food Chem. 2008;56(10):3532-7.

S11. Luo C, Yi B, Fan W, Chen K, Gui L, Chen Z, et al. Enhanced angiogenesis and astrocyte activation by ecdysterone treatment in a focal cerebral ischemia rat model. Acta Neurochir Suppl. 2011;110(Pt 1):151-5.

S12. Sundaram R, Naresh R, Shanthi P, Sachdanandam P. Efficacy of 20-OH-ecdysone on hepatic key enzymes of carbohydrate metabolism in streptozotocin induced diabetic rats. Phytomedicine. 2012;19(8-9):725-9.

S13. Foucault AS, Even P, Lafont R, Dioh W, Veillet S, Tomé D, et al. Quinoa extract enriched in 20-hydroxyecdysone affects energy homeostasis and intestinal fat absorption in mice fed a high-fat diet. Physiol Behav. 2014;128:226-31.

S14. Montes J, Goodwin AM, McDermott MP, Uher D, Hernandez FM, Coutts K, et al. Diminished muscle oxygen uptake and fatigue in spinal muscular atrophy. Ann Clin Transl Neurol. 2021;8(5):1086-95.

S15. Gorelick-Feldman J, Cohick W, Raskin I. Ecdysteroids elicit a rapid Ca2+ flux leading to Akt activation and increased protein synthesis in skeletal muscle cells. Steroids. 2010;75(10):632-7.

S16. Baker BF, Lot SS, Condon TP, Cheng-Flournoy S, Lesnik EA, Sasmor HM, et al. 2'-O-(2-Methoxy)ethyl-modified anti-intercellular adhesion molecule 1 (ICAM-1) oligonucleotides selectively increase the ICAM-1 mRNA level and inhibit formation of the ICAM-1 translation

initiation complex in human umbilical vein endothelial cells. J Biol Chem. 1997;272(18):11994-2000.
S17. Dilda P, Latil M, Didry-Barca B, On S, Serova M, Mamchaoui K, et al. BIO101 demonstrates combined beneficial effects on skeletal muscle and respiratory functions in a mouse model of Duchenne muscular dystrophy. 24th WMS Congress; 1-5th Oct; Copenhagen, Denmark: Neuromuscular Disorders, 29, S1, S158, abstract P311; 2019.
S18. Chali F, Desseille C, Houdebine L, Benoit E, Rouquet T, Bariohay B, et al. Long-term exercise-specific neuroprotection in spinal muscular atrophy-like mice. J Physiol. 2016;594(7):1931-52.
S19. Enjin A, Rabe N, Nakanishi ST, Vallstedt A, Gezelius H, Memic F, et al. Identification of novel spinal cholinergic genetic subtypes disclose Chodl and Pitx2 as markers for fast motor neurons and partition cells. J Comp Neurol. 2010;518(12):2284-304.
S20. Wadman RI, Vrancken AF, van den Berg LH, van der Pol WL. Dysfunction of the neuromuscular junction in spinal muscular atrophy types 2 and 3. Neurology. 2012;79(20):2050-5.
S21. Kong L, Wang X, Choe DW, Polley M, Burnett BG, Bosch-Marcé M, et al. Impaired synaptic vesicle release and immaturity of neuromuscular junctions in spinal muscular atrophy mice. J Neurosci. 2009;29(3):842-51.
S22. Biondi O, Grondard C, Lecolle S, Deforges S, Pariset C, Lopes P, et al. Exercise-induced activation of NMDA receptor promotes motor unit development and survival in a type 2 spinal muscular atrophy model mouse. J Neurosci. 2008;28(4):953-62.
S23. Aagaard P, Simonsen EB, Andersen JL, Magnusson P, Dyhre-Poulsen P. Increased rate of force development and neural drive of human skeletal muscle following resistance training. J Appl Physiol. 2002;93(4):1318-26.
S24. Mentis GZ, Blivis D, Liu W, Drobac E, Crowder ME, Kong L, et al. Early functional impairment of sensory-motor connectivity in a mouse model of spinal muscular atrophy. Neuron. 2011;69(3):453-67
S25. Rose FF, Jr., Mattis VB, Rindt H, Lorson CL. Delivery of recombinant follistatin lessens disease severity in a mouse model of spinal muscular atrophy. Hum Mol Genet. 2009;18(6):997-1005.
S26. Delbono O, Meissner G. Sarcoplasmic reticulum Ca2+ release in rat slow- and fast-twitch muscles. J Membr Biol. 1996;151(2):123-30.
S27. Biondi O, Lopes P, Desseille C, Branchu J, Chali F, Ben Salah A, et al. Physical exercise reduces cardiac defects in type 2 spinal muscular atrophy-like mice. J Physiol. 2012;590(22):5907-25.
S28. Latil M, Dilda P, Lafont R, Veillet S. Phytoecdysones and the derivatives for use in the treatment of impaired lung function. Biophytis. Patent WO2020187679
S29. Sullivan RM, Hofer MA, Brake SC. Olfactory-guided orientation in neonatal rats is enhanced by a conditioned change in behavioral state. Dev Psychobiol. 1986;19(6):615-23.
